# Integrative Metatranscriptomic Analysis Reveals Disease-specific Microbiome–host Interactions in Oral Squamous Cell Carcinoma

**DOI:** 10.1158/2767-9764.CRC-22-0349

**Published:** 2023-05-08

**Authors:** Vinay Jain, Divyashri Baraniya, Doaa E. El-Hadedy, Tsute Chen, Michael Slifker, Fadhl Alakwaa, Kathy Q. Cai, Kumaraswamy N. Chitrala, Christopher Fundakowski, Nezar N. Al-Hebshi

**Affiliations:** 1 Oral Microbiome Research Laboratory, Department of Oral Health Sciences, Maurice H. Kornberg School of Dentistry, Temple University, Philadelphia, Pennsylvania.; 2 Low level Radiation Research Section, Radiation Biology & Health Sciences Division, Bhabha Atomic Research Centre, Mumbai, India.; 3 Department of Microbiology, Forsyth Institute, Cambridge, Massachusetts.; 4 Biostatistics and Bioinformatics Facility, Fox Chase Cancer Center, Philadelphia, Pennsylvania.; 5 Department of Internal Medicine, Nephrology Division, University of Michigan, Ann Arbor, Michigan.; 6 Histopathology Facility, Fox Chase Cancer Center, Philadelphia, Pennsylvania.; 7 Fels Cancer Institute for Personalized Medicine, Lewis Katz School of Medicine, Temple University, Philadelphia, Pennsylvania.; 8 Thomas Jefferson University, Philadelphia, Pennsylvania.; 9 Cancer Prevention and Control Program, Fox Chase Cancer Center, Temple University Health System, Philadelphia, Pennsylvania.

## Abstract

Studies on the microbiome of oral squamous cell carcinoma (OSCC) have been limited to 16S rRNA gene sequencing. Here, laser microdissection coupled with brute-force, deep metatranscriptome sequencing was employed to simultaneously characterize the microbiome and host transcriptomes and predict their interaction in OSCC. The analysis involved 20 HPV16/18-negative OSCC tumor/adjacent normal tissue pairs (TT and ANT) along with deep tongue scrapings from 20 matched healthy controls (HC). Standard bioinformatic tools coupled with in-house algorithms were used to map, analyze, and integrate microbial and host data. Host transcriptome analysis identified enrichment of known cancer-related gene sets, not only in TT versus ANT and HC, but also in the ANT versus HC contrast, consistent with field cancerization. Microbial analysis identified a low abundance yet transcriptionally active, unique multi-kingdom microbiome in OSCC tissues predominated by bacteria and bacteriophages. HC showed a different taxonomic profile yet shared major microbial enzyme classes and pathways with TT/ANT, consistent with functional redundancy. Key taxa enriched in TT/ANT compared with HC were *Cutibacterium acnes*, *Malassezia restricta*, Human Herpes Virus 6B, and bacteriophage Yuavirus. Functionally, hyaluronate lyase was overexpressed by *C. acnes* in TT/ANT. Microbiome-host data integration revealed that OSCC-enriched taxa were associated with upregulation of proliferation-related pathways. In a preliminary *in vitro* validation experiment, infection of SCC25 oral cancer cells with *C. acnes* resulted in upregulation of MYC expression. The study provides a new insight into potential mechanisms by which the microbiome can contribute to oral carcinogenesis, which can be validated in future experimental studies.

**Significance:**

Studies have shown that a distinct microbiome is associated with OSCC, but how the microbiome functions within the tumor interacts with the host cells remains unclear. By simultaneously characterizing the microbial and host transcriptomes in OSCC and control tissues, the study provides novel insights into microbiome-host interactions in OSCC which can be validated in future mechanistic studies.

## Introduction

Oral squamous cell carcinoma (OSCC) is the predominant malignancy of the oral cavity with poor prognosis and a 5-year survival rate of less than 50% (1, 2), resulting in more than 175,000 deaths annually (3). The tongue is the most affected subsite of the oral cavity (4). Use of various forms of tobacco and alcohol consumption is the major risk factors of OSCC, accounting for nearly 74% of cases in Western countries (5). A small fraction of OSCC cases (2%–6%) will also possess high-risk human papillomavirus (HPV) strains though the potential causal role of HPV in OSCC has not been clearly demonstrated to the same extent as in the oropharynx (6, 7). Recently, there has been increasing interest in the role of the microbiome in cancer in general including OSCC (8–11).

A plethora of studies have been carried out to characterize the microbiome associated with OSCC in a variety of samples including surface swabs, oral rinse, unstimulated saliva, and tumor biopsies—which we comprehensively reviewed elsewhere (8, 9). While these studies demonstrate that OSCC-associated microbiome is significantly different from health-associated microbiome, the results do not reveal that a particular species or consortium is consistently enriched in OSCC samples across all patient cohorts. While these inconsistencies may have been a result of methodologic variations among the studies, they can probably be explained by functional redundancy: the fact that different species can serve the same function within microbial communities (12). In fact, Tian and colleagues (13) have recently shown that the gene composition and functional capacity of a microbiome is more conserved as compared with its taxonomic composition.

Consistently, we have recently identified different species in association with OSCC in two cohorts using 16S sequence-based compositional analysis; however, applying functional prediction analysis, we found proinflammatory microbial attributes to be enriched in both cohorts (14, 15). Similarly, a pilot study by Yost and colleagues (16) using metatranscriptomic approach (sequencing of mRNA transcripts from all organisms in a sample) revealed that OSCC-associated microbiomes have similar functional signatures despite differences in their taxonomic composition. Together, these findings provide evidence for microbial functional redundancy and highlight the importance of functional analysis in assessing the role of the microbiome in OSCC.

Metatranscriptome analysis is one approach to study the functional activity of a microbiome (17). Compared with 16S rRNA gene sequencing, which has been the predominant microbiome analysis method so far, metatranscriptomics captures only viable, transcriptionally active species, allows identification of all types of microorganisms in the sample (bacteria, archaea, fungi, and viruses) and provides higher taxonomic resolution. In addition, because samples will usually include host cells, metatranscriptomics provides an opportunity to simultaneously study the microbiome and host transcriptome and their potential interaction. To the best of our knowledge, the study by Yost and colleagues (16) is the only study so far that applied metatranscriptomics to OSCC. The study involved analysis of oral swabs collected from 4 patients with OSCC and 4 HC and was limited to assessment of the bacterial transcriptome; other microbial kingdoms and the host transcriptome were not evaluated.

In this first-of-kind study, we have employed laser microdissection (LMD) coupled with metatranscriptome sequencing at unprecedented depth (brute-force deep sequencing) to characterize the composition and function of the multi-kingdom microbiome within OSCC tissues and its association with the transcriptional activity of the host to provide novel insights into microbiome–host interactions in OSCC.

## Materials and Methods

**FIGURE 1:**
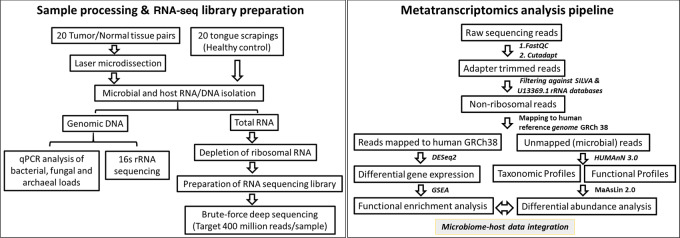
A flow chart of study design and procedures. Left: Study groups, sampling, sample processing, qPCR, RNA library preparation, and sequencing. Right: Bioinformatic analysis pipeline used to map, analyze and integrate the host and microbiome sequencing data.

An overview of study design and workflow is given in Fig. 1; the details are provided in the sections below. The study was approved by the Institutional Review Boards at Temple University (Philadelphia, PA; # 25808) and Thomas Jefferson University (Philadelphia, PA; #19D.270). The study was conducted in accordance with Declaration of Helsinki; a written informed consent was obtained from all prospectively recruited subjects.

### Subject Population and Samples

Frozen OSCC tumor and adjacent normal tissue pairs (abbreviated thereafter as TT and ANT, respectively) were obtained from the Biosample Repository Facility at Fox Chase Cancer Centre and the Pathology Biorepository Shared Service at the University of Maryland, Baltimore, MD. To minimize heterogeneity, the samples were restricted to cancer of the mobile tongue (ICD-10 code C02). Out of 50 tissue pairs initially obtained, only 20 pairs were found by histopathologic evaluation to be suitable for microdissection (next section). All cases were HPV16/18-negative as confirmed by PCR, and all except one were treatment naïve (1 subject had received radiotherapy before resection). As an additional control group, deep epithelial tongue scrapings were obtained prospectively from 20 age-, race-, and sex-matched HC given the following inclusion criteria: no evidence of malignancy and premalignant lesions, no signs of acute and/or chronic oral infections including severe gingivitis and/or periodontitis, no history of antibiotic and/or antifungal intake in the last 3 months and no history of endocarditis and/or valve issue, and no history of smoking. The tongue scrapings were collected from the dorsal surface, after drying with a cotton roll, using 10 heavy strokes with a 7 mm loop-type dermatologic curette (Acuderm Inc), which has been shown to capture sufficient samples for RNA analysis (18, 19). The scrapings were immediately placed in RNAlater (Thermo Fisher Scientific) and stored at −80°C. Demographic details and clinical characteristics of the study subjects are shown in Supplementary Table S1.

### Histopathologic Examination and LMD

Frozen TT and ANT samples were embedded in optimal cutting temperature medium and 8-μm-thick cryosections were cut using cryostat microtome. RNase free environment was maintained during all the steps. The sections were stained with hematoxylin and eosin for histopathologic evaluation and grading. On the basis of histopathologic review, 21–30 additional sections were cut for each tissue and placed on PEN membrane glass slides (Thermo Fisher Scientific) for LMD. The sections were processed and stained using Histogene Staining solution (Thermo Fisher Scientific) as per the manufacturer's instructions and sequentially dehydrated in 70%, 95%, and 100% alcohol, before air drying for 5 minutes at room temperature. All the solutions were treated with 1x ProtectRNA RNase inhibitor solution (Sigma-Aldrich) to prevent RNase contamination. LMD was performed using Leica LMD6500 gravity, contact-free collection system (Leica Microsystems). The desired areas (tumor cells and adjacent normal epithelium) were carefully marked under 5× magnification and captured in RNAlater placed on the cap of 0.5 mL PCR tube. Between 3 and 6 sections were captured per cap and multiple tubes were used to collect tissue from each sample to minimize capture time and thus RNA degradation. The microdissected sections in RNAlater were stored at −80°C until further processing. Representative images of microdissected tissues are shown in Supplementary Fig. S1.

### DNA and RNA Extraction

DNA and RNA were extracted using AllPrep DNA/RNA Micro kit (Qiagen), including a bead beating step to ensure lysis of microbial cells. Briefly, the tissue samples stored in RNAlater were thawed at 37°C, pelleted by adding equal volume of PBS and spinning at 5,000 rpm for 5 minutes, and resuspended in 600 μL of RLT plus lysis solution. The lysate was transferred into DNase/RNase free tubes containing 200 mg 100-μm zirconium beads (Molecular biology grade, PFMB 100-100-12, OPS Diagnostics), and bead beaten at 6 m/s for 1 minute at room temperature using FastPrep FP100A instrument (MP Biomedicals). The lysate was used to sequentially isolate DNA and RNA as per manufacturer's instructions. For RNA, in-column DNase treatment was done using RNase-Free DNase Set (Qiagen). Aliquots of RNAlater were used as extraction negative control. The purity of RNA and DNA was assessed by measuring 260/280 ratio using Nanodrop (Thermo Fisher Scientific) and quantity was measured using Qubit RNA HS Assay Kit and Qubit dsDNA HS Assay Kit (Thermo Fisher Scientific), respectively. The RNA integrity (RIN) and size distribution was assessed using Agilent RNA 6000 Pico Kit on Bioanalyzer 2100 (Agilent Technologies). DNA and RNA concentrations and RIN numbers for the samples are presented in Supplementary Data S1.

### Determination of Microbial Kingdom Loads

DNA isolated from the samples was used for determination of bacterial, archaeal, and fungal loads by qPCR. Universal primer pairs targeting bacteria (341F and R806), archaea (ARC344F and Arch806R), and fungi (*ITS1-30F*, *ITS1-217R*; refs. 20, 21) were used; the sequences are listed in Supplementary Table S2. Genomic DNA from *Haemophilus parainfluenzae* (NCTC 10665, Public Health England), *Methanobrevibacter oralis* (DSM 7256, DSMZ), and *Candida albicans* (CAI4 laboratory strain) was used as control for the bacterial, archaeal, and fungal assays, respectively. The PCR efficiency for each primer pair was derived from the standard curve prepared with at least five serial dilutions of control DNA (Supplementary Fig. S2). The PCR reaction mix (20 μL) contained 5 ng sample DNA, 1X PowerUp SYBR Green Master Mix (Thermo Fisher Scientific), 1 μmol/L of each primer (for bacteria), 0.5 μmol/L of each primer (for archaea), and 0.125 μmol/L of each primer (for fungi). The cycling conditions were as follows: 50°C for 2 minutes for uracil-dna glycosylase activation, 95°C for 2 minutes for polymerase activation followed by 45 cycles of denaturation at 95°C for 15 seconds and annealing and/or extension at 60°C (bacterial and archaeal primers) and 62°C (fungal primers). All qPCR reactions were carried out in triplicates on a Quantstudio 3.0 thermal system (Thermo Fisher Scientific). No template, extraction, and positive controls were added in each run. Abundance of each kingdom was normalized to human β-actin gene as described previously (22).

### rRNA Depletion, Library Preparation, and Sequencing

Bacterial and human rRNA were depleted from total RNA using NEBNext rRNA Depletion Kits E7850 and E7400 (New England Biolabs), respectively. A cocktail of human and bacterial depletion solutions was used in the ratio of 2:1. Depleted RNA was purified using Agencourt RNA clean XP beads (Beckman Coulter) and used for preparation of RNA sequencing (RNA-seq) libraries using NEBNext Ultra II Directional RNA Library Prep (New England Biolabs) as per the manufacturer's protocol. The RNA fragmentation time was optimized and adjusted according to RIN no. Libraries were labeled with unique indexes for multiplexing using NEBNext Multiplex Oligos for Illumina (New England Biolabs). The final libraries were quantified using Qubit dsDNA HS Assay Kit on Qubit 3.0 fluorimeter. The library quality and size distribution were assessed using Agilent High sensitivity DNA kit on Bioanalyzer 2100. Library concentrations are presented in Supplementary Data S1. The 60 libraries were pooled in groups of 10 and sequenced using 2 × 100 CoolMPS chemistry on DNBSEQ-T7 platform (BGI) with a target depth of 400 million paired reads per sample (brute-force deep sequencing).

### Mapping of Human and Ribosomal Sequences

Paired-end FASTQ files were quality checked using FastQC (RRID:SCR_014583 https://www.bioinformatics.babraham.ac.uk/projects/fastqc/) and MultiQC (23), and NEBNext adapter content was trimmed with Cutadapt (24). Trimmed FASTQ files were aligned separately to human reference GRCh38, the SILVA large subunit rRNA database (v138), and the human ribosomal DNA complete repeating unit U13369.1 using the STAR aligner (RRID:SCR_004463; ref. 25). A custom script was used to identify reads aligning to various combinations of the three references and reads which were unmapped to any of the three references were saved as FASTQ files for mapping to microbial sequences (see below).

### Host Differential Gene Expression and Pathway Analysis

Reads aligning to GRCh38 were quantified at the gene-level using *htseq-count* [HTSeq] (mode = “union”; ref. 26). Differential expression analysis was carried out with *DESeq2* (RRID:SCR_000154; ref. 27) for the following comparisons (each involving 40 samples): (i) TT versus ANT (paired comparison), (ii) TT versus HC, and (iii) ANT versus HC. For each pairwise comparison, any gene with fewer than 50 total counts across the 40 samples was excluded prior to DESeq2 analysis. Subsequently, a ranked list of differentially expressed genes (DEG) for each comparison (ranked by Wald statistic generated by *DESeq2*) was used as input to gene set enrichment analysis (GSEA, *Preranked* method; ref. 28) to explore whether phenotypic differences were significantly related to known functional gene sets. The hallmark gene set collections from Molecular Signatures Database (MSigDB) 7.1 was used for this purpose (29).

### Microbiome Data Analysis

Reads not mapping to any of the three reference databases above were further processed with KneadData (30) to further remove any remaining human and rRNA sequences. KneadData-cleaned, unmapped reads were then analyzed with HUMAnN 3.0 for microbial profiling (31). HUMAnN 3.0 uses MetaPhlAn 3.0 (32) for taxonomic profiling, including viruses. For functional profiling, it uses Bowtie2 (33) for nucleotide-level searches against ChocoPhlAn 3 database (34) followed by translated search of yet unmapped reads against UniRef90 protein reference database (35) using DIAMOND (36). The individual sample gene lists generated are then combined and regrouped using different functional annotations. In this study, we used enzyme classes (EC) and metabolic pathways based on MetaCyc database (RRID:SCR_007778; ref. 37). Ultimately unmapped reads, and taxa/functional features present in ≤10% of the samples were excluded before the profiles were centered log ratio (CLR) transformed (38) to account for compositionality of the data (39). MaAsLin2 (Microbiome Multivariable Associations with Linear Models) package in R (40) was employed to identify differentially abundant features between the groups (TT, ANT, and HC), accounting for the paired nature of the comparison in the TT versus ANT contrast; FDR according to Benjamini and colleagues (41) was set to 0.05. Principle component analysis (PCA) was performed using Phyloseq (42) and “microbiome” (43) R packages. Permutational multivariate ANOVA (PERMANOVA) test was performed using vegan package in R (44).

### Microbiome-host Data Integration

Unsupervised Spearman rank correlation was performed between the host genes’ expression levels (DESeq2 transformed) and CLR-transformed microbial features (genera and EC). Significant correlations were defined as those with absolute correlation coefficient (*r*_s_) ≥ 0.6 and FDR ≤0.05. For each microbial feature, the correlating genes, ranked by *r*_s_, were subjected to GSEA (28) to identify pathways potentially modulated by that feature. The results were visualized with Cytoscape Automation (45) with microbes and host pathways as nodes and enrichment scores as edges. We also employed MOFA (Multi Omics Factor Analysis) (46) as another method to integrate the host and microbiome data. MOFA reduces multiple high-dimensional data into a small number of factors that captures biological variation in the data, and then measure the contribution of omic sets to each factor, and ability of each factor to discriminate between the study subgroups.

### Validation Assays

For the host transcriptome, expression of seven highly DEGs (*MMP13*, *CA9*, *ROS1*, *KRT4*, *CRNN*, and *SPRR3*) was validated with quantitative, reverse-transcription PCR (qRT-PCR). The assays were performed using predesigned gene-specific primers/probe sets in customized TaqMan Array Standard Plates (Thermo Fisher Scientific). Specifically, EXPRESS One-Step Superscript qRT-PCR Kit (Thermo Fisher Scientific) was used in 20 μL reactions as recommended by the manufacturer, using 10 ng total RNA as template on Quantstudio 3.0 thermal cycler (Applied Biosystems). The following cycling conditions were used: cDNA synthesis for 15 minutes at 50°C, initial denaturation at 95°C for 2 minutes and 40 cycles of denaturation at 90°C for 15 seconds and extension at 60°C for 1 minute with data collection at the end of each extension step. All the genes were studied in triplicates and normalized with RPL30 as endogenous control.

### 16S rRNA Gene-based Microbial Profiling

To supplement the information obtained from RNA-seq, microbial composition was also assessed by 16S rRNA gene sequencing of the DNA extracts. Library preparation, sequencing, and bioinformatics analysis were done as described previously (14).

### Contamination Control

Extraction negative controls were included for both RNA and 16S rRNA gene sequencing. In the former, no detectable levels of RNA were found using Qubit RNA High Sensitivity kit, so no further processing (i.e., library preparation) could be performed. For 16S rRNA gene sequencing, no amplifiable DNA was detected in the extraction negative controls by PCR, but they were still submitted for sequencing. We then performed manual filtration of probable contaminants based on (i) established knowledge of the microbial taxa typically found in the oral cavity (HOMD.org), (ii) contaminants found in the sequenced negative control as well as those reported in the literature [as in Salter and colleagues 2014 (47)], and (iii) comparing 16S and RNA-seq profiles. All three points together were considered when deciding about taxa to filter out, that is, they were not sequential filtering steps. For example, *Microbacterium* and *Sphingomonas* represented 27% and 18% of the reads in the 16S data from TT and ANT samples, respectively. These genera have been reported as negative control contaminants, are not typical members of the oral microbiome, and were not detected in the RNA-seq data (i.e., do not represent live/transcriptionally active bacteria). Therefore, they were filtered out from the 16S profiles. In contrast, while genus *Streptococcus* has been reported as a negative control contaminant, it is also a major member of the oral microbiome, so it was not removed. Other known contaminants found in high abundance in the extraction negative controls by 16S sequencing included *Bradyrhizobium, Stenotrophomonas*, *Hyphomicrobium*, and *Escherichia*, and again were not observed in the RNA-based profiles. Together, the results provided a proof that the RNA-seq data were free from DNA contamination.

### Availability of Data and Materials

The data are made available for secondary use through the database of Genotypes and Phenotypes (dbGaP), accession number phs002678.v1.p1. However, due to consent constraints, the human sequences from the HC data were filtered out and cannot be shared for future research.

## Results

### Sequencing Statistics

Ultra-deep sequencing of the 60 samples generated a total of approximately 23 billion 2 × 100 bp reads (∼10TB of data), with an average of 398,569,093 ± 62,427,540 reads per sample. Per group, the average sequencing depth was 422,326,741 ± 70,776,305 for TT, 384,960,281 ± 55,224,182 for ANT, and 388,420,257 ± 56,083,238 for HC. At the Star aligner step, TT and ANT samples showed a similar pattern with approximately 97% of the reads mapping to GRCh38, approximately 0.7% mapping to SILVA and U13369.1 combined, and approximately 2.0% remaining unmapped (Supplementary Fig. S3). In the HC group, on the other hand, only 23.4% of the reads mapped to GRCh38, while 10% mapped to the rRNA databases and 67% remained unmapped. Processing the unmapped reads with kneadData removed an average of 84% and 9% of the reads from the TT/ANT and HC groups, respectively, as contaminating sequences (i.e., human and ribosomal sequences that did not meet STAR's mapping parameters). Eventually, an average of 1.3M and 250M reads from the TT/ANT and HC groups, respectively, were available as input for HUMAnN analysis. Detailed sequencing and mapping statistics are presented in Supplementary Data S2.

### Host Transcriptome Analysis Confirms Known Cancer-associated Genes and Pathways and Reveals Field Cancerization in Tumor-adjacent Normal Tissue

**FIGURE 2:**
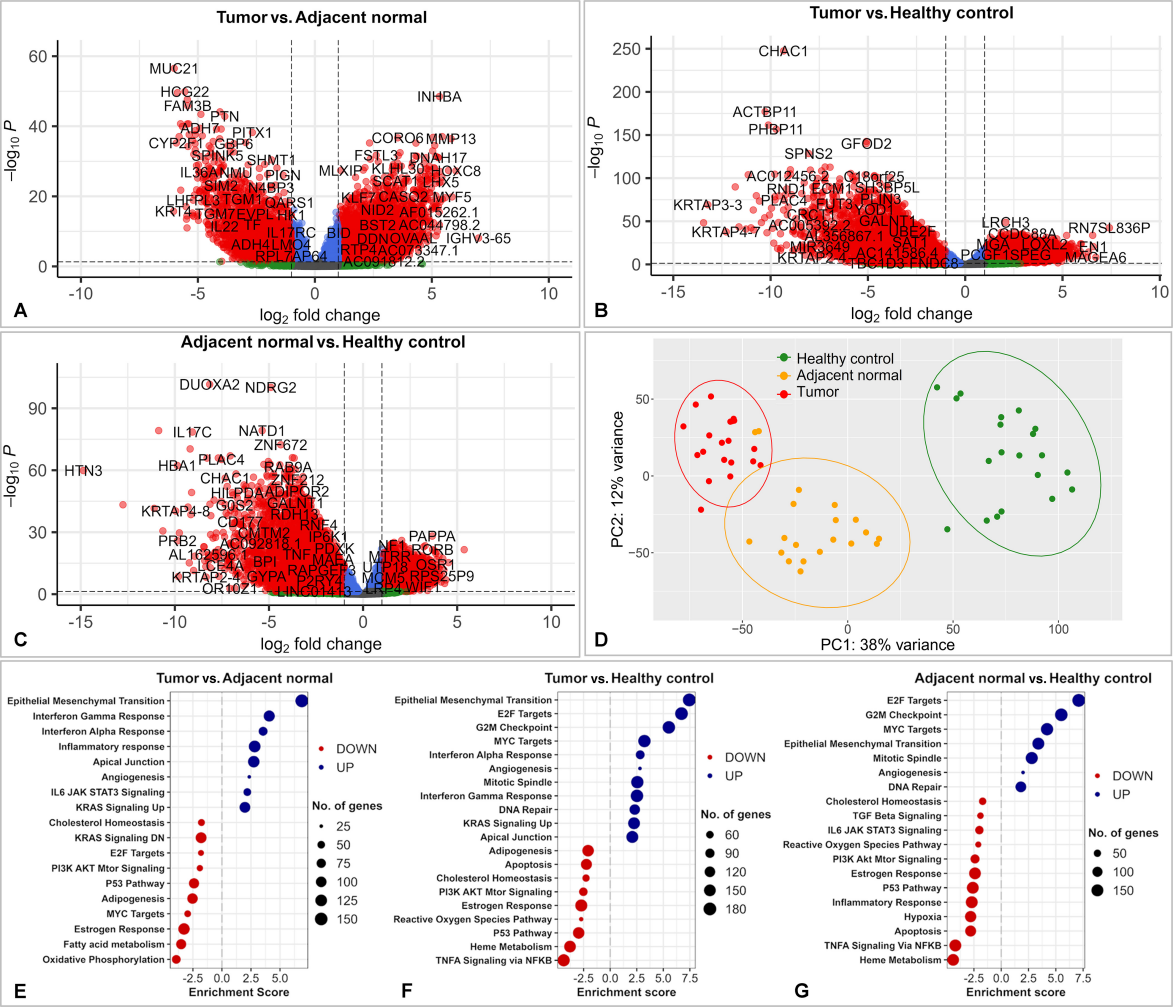
Host transcriptome. Sequences were mapped to human reference GRCh38 and quantified at the gene level using STAR aligner and HTSeq, respectively. *DESeq2* was used to identify DEGs which are depicted in volcano plots for tumor versus adjacent normal (paired comparison; **A**), tumor versus HC (**B**), and adjacent normal versus HC (**C**)—the color code in the volcano plots corresponds to the fold change and FDR cutoffs (2 and 0.05, respectively). **D,** A PCA plot based on the most variable 1,500 DEGs, generated using “plotPCA" function from DESeq2 R/Bioconductor package. Lists of DEGs preranked by Wald statistic were used as input for GSEA to identify upregulated and downregulated pathways in each contrast (**E–G**) based on Hallmark gene sets (MSigDB 7.1).

A total of 410,397,495 (TT), 375,786,295 (ANT), and 91,631,306 (HC) reads mapped to the human reference GRCh38, which identified 54,221, 52,454 and 51,716 coding, noncoding and pseudogenes, respectively, 56,962 in total. Filtration, normalization, and differential gene expression analysis was carried using DESeq2 in three contrasts: TT versus ANT, TT versus HC, and ANT versus HC. The output of these pairwise analyses, including fold change, FDR, and Wald statistic are presented in Supplementary Data S3. Applying cutoffs of ≥2.0-fold change and FDR ≤ 0.05 identified 8,640 DEGs in TT versus ANT (5,186 upregulated and 3,454 downregulated; Fig. 2A), 17,991 DEGs in TT versus HC (10,400 upregulated and 7,591 downregulated; Fig. 2B) and 15,375 DEGs in ANT versus HC (8,436 upregulated and 6,939 downregulated; Fig. 2C). PCA based on the top 1,500 most variable DEGs showed clear separation between the three groups (Fig. 2D). Results of the validation qRT-PCR assays for six DEGs were consistent with those obtained with RNA-seq, although the latter tended to underestimate the magnitude of fold change (Supplementary Table S3).

The details of GSEA results for all three contrasts are provided in Supplementary Data S4. In the TT versus ANT contrast, *INHBA* (Inhibin Subunit Beta A) stood out as the most significantly upregulated gene and contributed to enrichment of the epithelial-to-mesenchymal transition (EMT), inflammatory response and KRAS pathways (Fig. 2E). Other highly upregulated genes as part of these pathways were matrix metalloproteinase, collagen, and growth factor genes (Table 1). Interferon alpha and gamma responses, IL6/JAK/STAT signaling, and angiogenesis were also upregulated in TT versus ANT while oxidative phosphorylation, fatty acid metabolism, adipogenesis, and P53 pathway were downregulated (Fig. 2E). The key genes involved in each of these pathways are presented in Table 1. Apart from the hallmark gene sets, it is worth mentioning that HOX genes belonging to all the four clusters (A, B, C, D), and the corresponding long noncoding RNAs (lncRNA) were also highly upregulated in the TT versus ANT contrast. Interestingly, proliferation-related gene sets E2F targets, MYC targets, and G_2_–M checkpoint were downregulated in the TT versus ANT contrast. In contrast, they were the top upregulated pathways in the TT versus HC as well as the ANT versus HC comparisons (Fig. 2F and G). Largely, the same set of genes was involved in both comparisons (Table 1). EMT pathway was also upregulated in the two contrasts but did not involve any MMPs as in the TT versus ANT contrast. Instead, mainly genes encoding extracellular matrix proteins and adhesion molecules were upregulated (Table 1). In the other direction, genes involved in heme metabolism, P53 pathway, and apoptosis were downregulated. Counterintuitively, TNFα signaling via NFκB and inflammatory response pathway were also downregulated. Overall, the results from the ANT versus HC contrast demonstrate presence of oncogenic changes in the ANT consistent with field cancerization.

**TABLE 1:** Key genes within significantly enriched host pathways

Tumor tissues compared with the adjacent normal tissues	
Pathway	Genes
***Upregulated***	
Epithelial-to-mesenchymal transition Inflammatory response KRAS signaling	*INHBA* Matrix metalloproteinases: *MMP1*, *MMP2*, *MMP3, MMP9, MMP10, MMP11,* and *MMP14* Collagen genes: *COL4A1*, *COL4A2*, *COL11A1*, *COL12A1,* and *CTHRC1* Growth factors: *TGFBI*, *IGF2,* and *CSF2* Others: *FSTL3* (Follistatin-like 3) and *ITGA5* (Integrin subunit α5)
Interferon alpha and gamma responses	IFN-induced proteins: *IFI27*, *IFIT1*, *IFI35*, *IFIT3,* and *ISG15*
IL6/JAK/STAT pathway	CXCL genes, *IL6*, *STAT1*, *TGFBI,* and *CSF2*
Angiogenesis	*VAV2*, *SPP1*, *COL5A2*, *PDGFA,* and *POSTN*
Others	HOX genes (clusters A, B, C, D) and (e.g., HOTAIR)
***Downregulated***	
Oxidative phosphorylation	*MPC1*, *ETFDH*, *VDAC2*, *ACADSB*, *ACAA1*, *ACADVL*, *RETSAT*
Fatty acid metabolism	*ADH7*, *HPGD*, *CBR3*, *ALDH3A1*, *ALDH3A2*
Adipogenesis	*CYP4B1*, *EPHX2*, *PPARG*, *ELOVL6*, *MGLL*
P53 pathway	*KLF4*, GLS2, *BAIAP2*, *CDKN2AIP*, and *EPS8L2*
**Tumor/Adjacent normal compared with HC**	
**Pathway**	**Genes**
***Upregulated***	
E2F targets	*STAG*, *PRIM2*, *SMC6*, *MRE11*, *PRKDC, CHEK2,* and *BRCA2*
MYC targets	*CBX3*, *DDX18*, *HSPD1*, *NOP56*, *MCM4,* and *NOLC*
G_2_–M checkpoint genes	*STAG1*, *CDC27*, *SRSF10*, *MYC*, *WRN*, *CENPE,* and *MNAT1*
Epithelial-to-mesenchymal transition	Extracellular matrix proteins and adhesion molecules*: DST*, *COL4A1*, *CDH6*, *PLOD3*, *GEM*, *LAMC1*, *MYLK*, *COLGALT1,* and *LAMA3*
***Downregulated***	
Heme metabolism	*FBXO34*, *HBB*, *BPGM*, *RHCE*, *ADIPOR1*, and *LMO2*
P53 pathway	*MXD1*, *TGFA*, *CDKN2AIP*, *HMOX1*, *SAT1*, *FOXO3*
Apoptosis	*EMP1*, *SQSTM1*, *HMOX1*, *BCL2L1*, *H1-0*, *IL18*, *CDKN1A* and *BCL10*
TNFα signaling via NFκB	*DUSP5*, *TNIP1*, *MXD1*, *IL23A*, *MAP2K3*, *IL1A*, *IL1B,* and *TNF*
Inflammatory response pathway	*MXD1*, *FFAR2*, *IRAK2*, *IL1A*, *SPHK1*, *RAF1,* and *CXCL8*

As secondary analysis, we also compared within the OSCC cases between samples with and without lymph nodes involvement. At an FDR cutoff of ≤0.05, only 227 DEGs were identified (50 at fold change ≥2.0); using a more lenient cutoff (0.2) increased the number to 1,311 DEGs (Supplementary Data S5). At the gene level, *FDCSP* (cancer cell migration and invasion), *KIR2DL3* (immune response), *SLC30A10* (antiapoptotic), *MAB21L2*, *PCDH9* (cell adhesion), *PEG3* (cell proliferation) were found to be highly expressed in the Lymph-node (LN)-positive samples. At the pathway level, MYC targets, E2F targets, MTORC1 and KRAS signaling were significantly enriched in LN-positive group (Supplementary Data S5; Supplementary Fig. S4).

### Low Abundance Yet Unique, Transcriptionally Active Multi-kingdom Microbiome in OSCC Tissues

**FIGURE 3:**
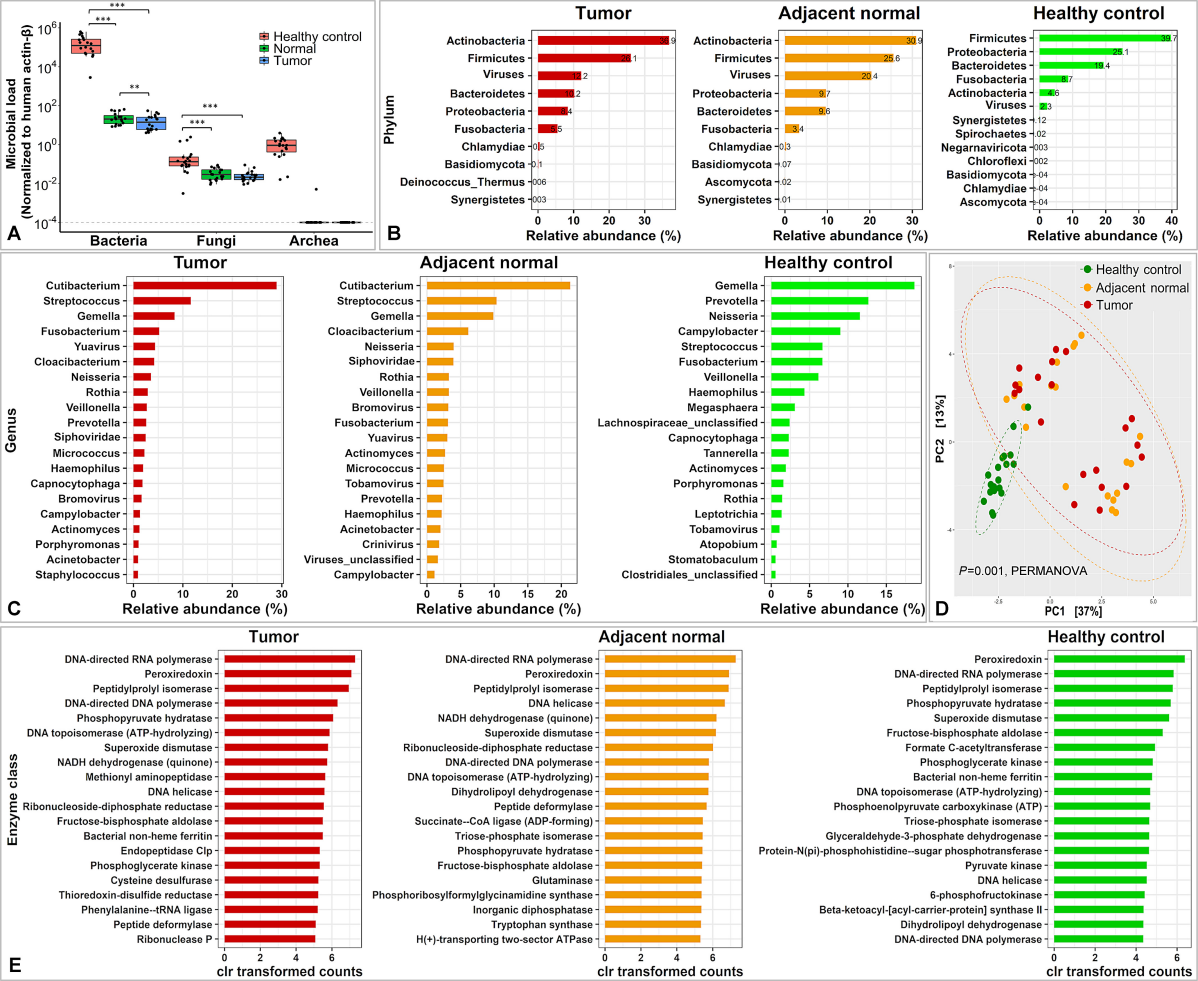
Microbial loads and transcriptional profiles. DNA extracted from the same cells as for RNA was used to determine bacterial, fungal, and archaeal loads in the samples relative to human β-actin gene by qPCR (**A**). Nonhuman, nonribosomal RNA sequences were used as input for HUMAnN 3.0 for microbial taxonomic and functional profiling. Microbial phyla (**B**) and top 20 genera (**C**) identified in each of the study groups ranked by their average transcriptional relative abundances. **D,** A PCA plot based on CLR-transformed genus-level profiles (created with Phyoseq and microbiome R packages). **E,** Top 20 expressed enzyme classes ranked by their average transcriptional abundances (CLR-transformed counts) in each sample type.

Loads of bacteria, fungi, and archaea normalized to human β-actin gene were determined by qPCR analysis of DNA extracted from the same cells as for RNA. Bacterial and fungal DNA were detected in all the samples; however, archaeal DNA was only detected in the HC (tongue scraping) group (Fig. 3A). As expected, the microbial loads were far higher in the HC (∼10^4^-fold and ∼15-fold for bacteria and fungi, respectively). The abundance of bacterial DNA in adjacent normal tissue was marginally but significantly higher than tumor (*P* ≤ 0.001); a similar trend was seen for fungal DNA, but the differences were not statistically significant.

Despite low abundance, analysis of nonhuman, nonribosomal RNA sequences with HUMAnN 3.0 pipeline identified a transcriptionally active, multi-kingdom microbiome in the samples. In the taxonomic profiling step by MetaPhlAn 3.0, a total 14 phyla, 165 genera, and 483 species were identified. The relative transcriptional abundances of each of these taxa in individual samples are presented in Supplementary Data S6. The average taxonomic profiles based on the 20 most abundant phyla and genera in each group are presented in Fig. 3B and C; the corresponding species-level profiles are presented in Supplementary Fig. S5. In the HC samples, the microbial transcriptome was dominated by the bacterial phyla Firmicutes, Proteobacteria, Bacteroidetes, Fusobacteria, and Actinobacteria (in this order of abundance). The TT and ANT groups had similar profiles with Actinobacteria being the most transcriptionally abundant, followed by Firmicutes and viruses; the latter accounted for a significant proportion of the transcripts (12.2% and 20.4%, respectively, compared with 2.3% in the HC samples). Fungal transcripts were identified in all three groups at very low abundance. At the genus level, *Gemella* was the most transcriptionally abundant in the HC group, followed with other typical oral genera including *Prevotella*, *Neisseria*, *Campylobacter*, *Streptococcus*, *Fusobacterium,* and *Veillonella*. On the other hand, while *Streptococcus, Gemella* and *Neisseria* were also among the top transcriptionally abundant taxa in the TT and ANT groups, *Cutibacterium* (predominantly *Cutibacterium acnes*) was the most abundant accounting for more than 20% of the transcripts on average (compared with <1% abundance in the HC). Also, *Cloacibacterium* (predominantly *Cloacibacterium normanens*) and bacteriophage Siphoviridae (including Yuavirus) were among the most abundant taxa. Yuavirus was predominantly represented by bacteriophage alpha proteobacterium JL001. In PCA (Fig. 3D), the HC group clustered separately from the TT and ANT groups (*P* = 0.001, PERMANOVA); however, the latter two groups did not differ and showed significant dispersion.

Following taxonomic profiling, the sequences were mapped to ChocoPhlAn 3 and UniRef90 databases which identified around an average of 23,556, 23,845, and 98,569 genes per sample in the TT, ANT, and HC groups, respectively. Rarefaction analysis of the number of microbial genes detected in each sample as a function of number of mapped reads (Supplementary Fig. S6) revealed that all samples reached saturation with a Good's coverage index of >99%. The gene lists were then regrouped and functionally annotated using MetaCyc database. The individual sample EC and metabolic pathway profiles are presented in Supplementary Data S7. The average functional profiles based on the top 20 ECs and top 20 pathways in each group are presented in Fig. 3E and Supplementary Fig. S7, respectively. Despite major differences in taxonomic profiles, the most abundant ECs and pathways were common to all three groups consistent with functional redundancy. Abundant ECs included those involved in DNA replication and transcription (DNA polymerase, DNA helicase, and RNA polymerase), response to oxidative stress (superoxide dismutase, peroxiredoxin, and thioredoxin-disulfide reductase), and metabolism (e.g., adolase, phosphoglycerate kinase, dihydrolipoyl dehydrogenase, and pyruvate kinase). At the pathway level, glycolysis, biosynthesis of nucleotides, and biosynthesis of peptidoglycan were the dominant pathways. PCA by ECs did not show separate clusters between the three groups, but the HC group formed a compact subcluster with little dispersion (Supplementary Fig. S8).

For comparison and validation, we also performed 16S profiling on DNA extracted simultaneously with the RNA. Statistically, the 16S and RNA-seq bacterial taxonomic profiles were overall significantly different (*P* = 01, PERNAMOVA) with the RNA-seq profiles showing more dispersion (Supplementary Fig. S9). Notably, *Gemella*, which was the most transcriptionally abundant genus in the HC group and third most abundant in the TT and ANT groups, did not show up among the 20 top abundant genera by 16S sequencing. Conversely, *Corynebacterium* was among the top genera in the TT/ANT samples but accounted for less than 0.5% of the transcripts in the RNA-seq data. Nevertheless, there were consistencies between the two methods, for example, *Cutibacterium* (formerly *Propionibacterium*) was the most abundant genus in the TT and ANT groups in both methods.

### Cutibacterium Acnes, Malassezia Restricta, Human Herpes Virus 6B, Nupapillomavirus, Bacteriophages, and Hyaluronate Lyase are Key Features Enriched in OSCC Tissues

**FIGURE 4:**
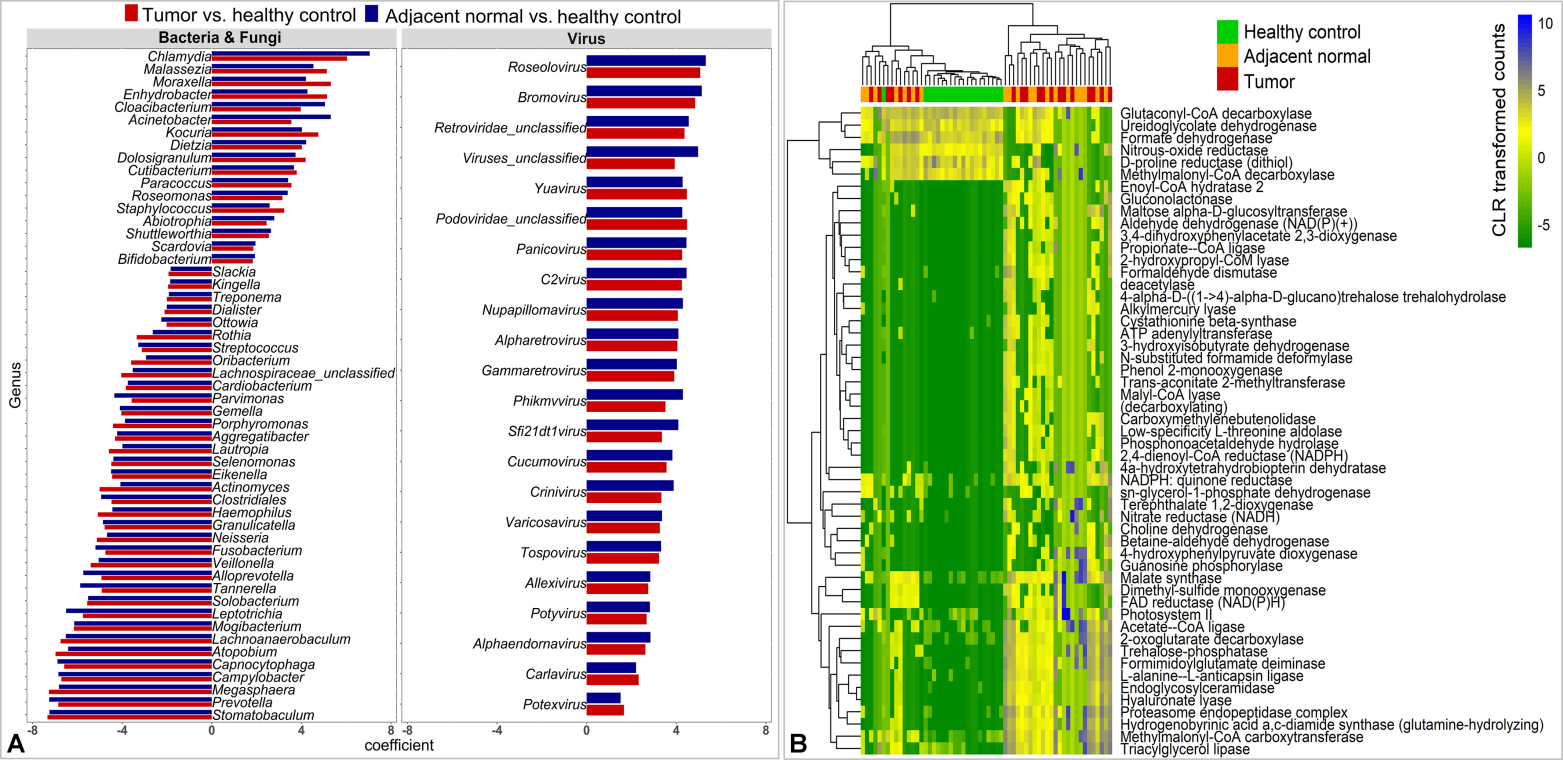
Differentially abundant microbial features. Taxonomic and functional profiles obtained with HUMAnN 3.0 were CLR transformed and differential abundance analysis was performed with MaAsLin2 setting FDR cutoff to 0.05. **A,** Bar plots of the top differentially abundant genera in the tumor versus HC and adjacent normal versus HC contrasts. **B,** A heatmap showing clustering of samples based on top differentially abundant enzyme classes. The plots were created with ggplot2 and pheatmap R packages.

Pairwise (TT vs. ANT, TT vs. HC, and ANT vs. HC) differential abundance analysis was performed with MaAsLin2 on CLR-transformed taxonomic (genus and species level) and functional profiles. The full results of this analysis in the form of lists of taxa and functional features and the corresponding coefficients and FDR values are provided in Supplementary Data S8 and S9. No significant differences in microbial profiles were found between the TT and ANT groups; however, the differences were dramatic for the TT versus HC and ANT versus HC contrasts, and for the most part, were similar between the two comparisons as seen in Fig. 4 (top differentially abundant genera and ECs) and Supplementary Fig. S10 (top differentially abundant species and pathways). Thirty-six bacterial genera that are commonly found in the oral cavity were transcriptionally more abundant in the tongue scrapings (HC group), including *Gemella*, *Prevotella*, *Neisseria*, *Campylobacter*, *Fusobacterium*, *Veillonella*, *Streptococcus*, *Haemophilus*, *Capnocytophaga*, *Tannerella*, *Actinomyces*, *Rothia,* and *Porphyromonas* but *Stomatobaculum* was the most differentially abundant (Fig. 4A). In the TT and ANT tissues, however, less common/typical oral bacteria were transcriptionally enriched including *Chlamydia*, *Moraxella*, *Enhydrobacter*, *Claocibacterium*, *Acinetobacter,* and *Cutibacterium*. Of these, *Cutibacterium* (predominantly *C. acnes*) was the most abundant and thus chosen for validation by qPCR (Supplementary Table S4) which showed consistent results (Supplementary Fig. S11). Besides bacteria, the fungus *Malassezia restricta* and several viruses were also transcriptionally more abundant in the TT and ANT groups. Enriched viruses can be grouped into human viruses (Roseolovirus represented by Human Herpes Virus 6B and Nupapillomavirus), bacteriophages (predominantly Siphoviridae, genus Yuavirus, species alpha proteobacterium JL001), plant viruses (e.g., Bromovirus) and retroviridae (Supplementary Table S5). The latter group was detected in very low abundance and included mainly Avian endogenous retrovirus EAV-HP.

**FIGURE 5:**
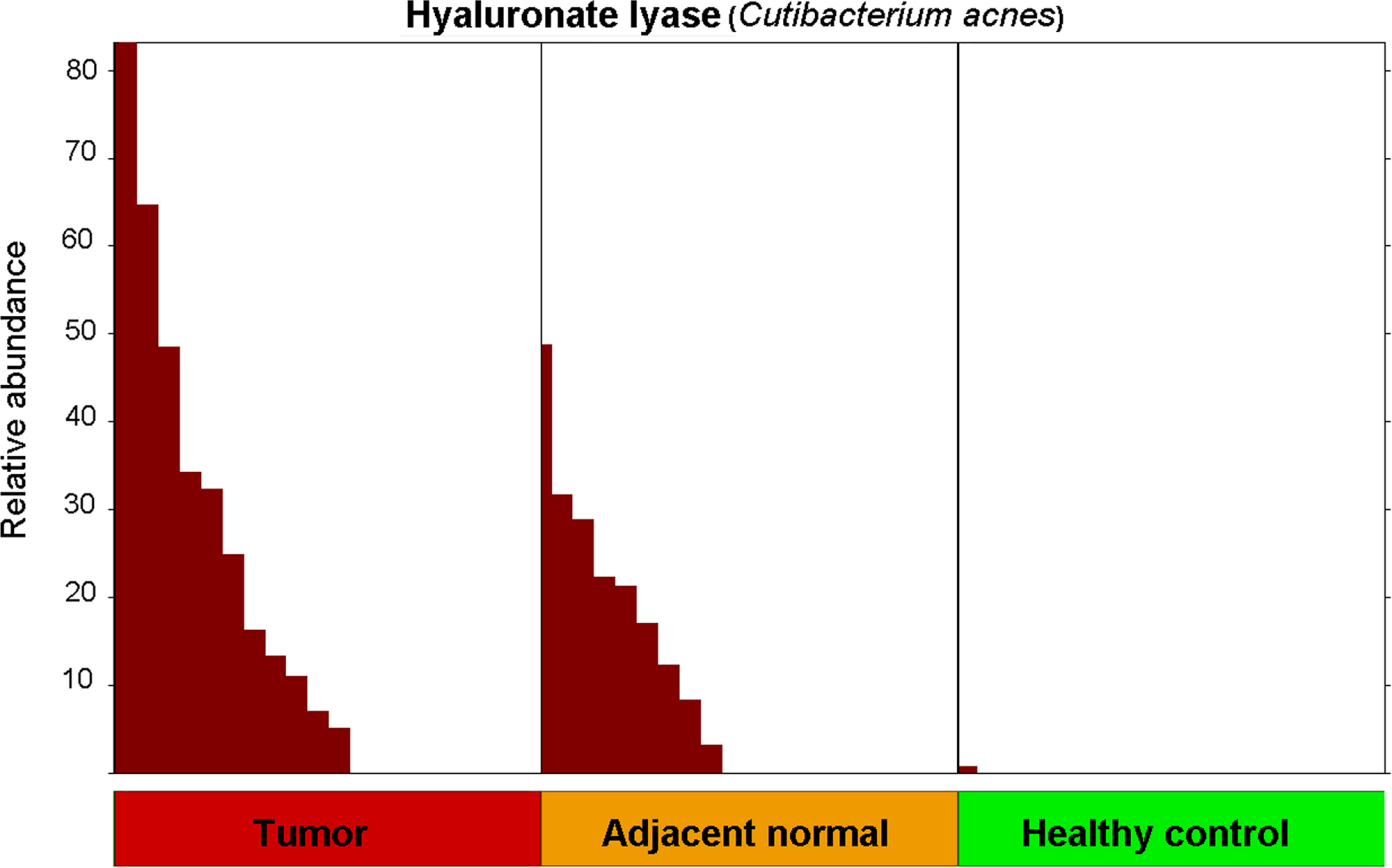
HL expression by *Cutibacterium acnes*. Relative abundance of HL transcripts in individual samples. The transcripts were exclusively contributed by *Cutibacterium acnes* based on HUMAnN 3.0 results.

Hierarchical clustering of samples by top differentially expressed ECs and pathways is presented in Fig. 4B and Supplementary Fig. S10B, respectively. On the basis of ECs, the analysis resulted in a cluster with all HC samples and two clusters with mixed TT and ANT samples, with the smaller of the two being closer to the HC cluster—roughly similar clusters were seen at the pathway level. Regardless of clustering, ECs that were overexpressed in most TT/ANT samples include methylmalonyl-CoA decarboxylase, trehalose-phosphatase, dimethyl-sulfide monooxygenase, malate synthase, triacylglycerol lipase, endoglycosylceramidase, proteasome endopeptidase complex, formimidoylglutamate deiminase, and hyaluronate lyase (HL). Of these, we found the latter to be of potential relevance as it has hyaluronic acid degrading properties and can contribute to extracellular matrix breakdown and consequently facilitate tumor invasion. On the basis of HUMAnN results, HL was exclusively contributed by *C. acnes* in the TT/ANT samples (Fig. 5).

### OSCC-associated Microbial Taxa Potentially Modulates Host Proliferation Pathways

**FIGURE 6:**
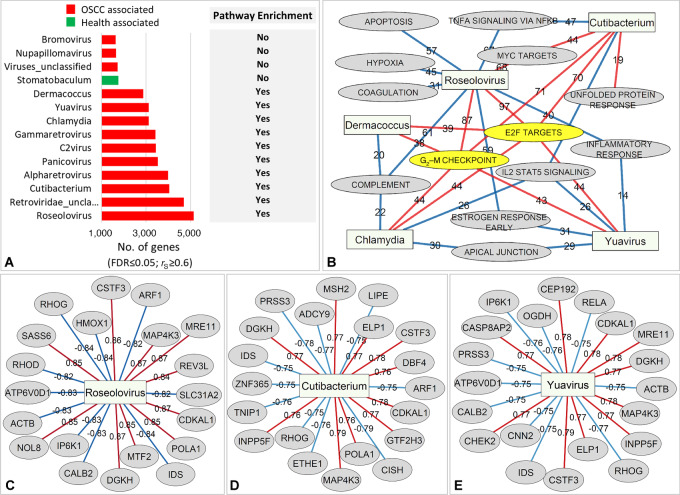
Microbiome-host data integration. Unsupervised Spearman rank correlation was performed between appropriately transformed host gene and microbial genus counts. Significant correlations were defined as those with absolute correlation coefficient (*r*_s_) ≥ 0.6 and FDR ≤ 0.05. For each microbial feature, the correlating genes, ranked by *r*_s_, were subjected to GSEA. **A,** Genera that correlated with >1,000 host genes and whether GSEA turned significant results for each genus. **B,** Interaction of selected genera with the host pathways based on GSEA results. Red edges denote activation while blue edges denote inhibition. For each edge, the number of genes involved is displayed. **C–E,** MSigDB genes with the highest correlations with Roseolovirus, *Cutibacterium* and Yuavirus, respectively. Red edges denote positive correlation while blue edges denote inhibition. For each edge, *r*_s_ is displayed.

To predict potential microbiome–host interactions, we performed unsupervised correlation analysis between the microbial features and host genes and then, for each feature, performed GSEA on significantly correlating genes to identify host pathways potentially modulated by that feature. The detailed outputs from these analyses are included in Supplementary Data S10–S12. Only OSCC-associated genera, including roseolovirus, *Cutibacterium*, retroviridae, *Chlamydia*, *Dermococcus*, Yuavirus showed substantial correlations (>3,000 genes each) and resulted in significant gene set enrichment (Fig. 6A and B). All these taxa showed association with upregulation of proliferation-related gene sets E2F targets and G_2_–M checkpoint. Roseolovirus and *Cutibacterium* were also correlated with upregulation of MYC targets. As examples, the 10 most positively correlated and 10 most negatively correlated MsigDB genes with Roseolovirus, Cutibacterium, and Yuavirus are presented in Fig. 6C–E. Functionally, three ECs, namely guanosine phosphorylase, terephthalate 1,2-dioxygenase, and nitrate reductase (NADH) were also associated with upregulation of proliferation-related pathways Supplementary Fig. S12A and S12B.

Data integration with MOFA reduced the variation in host and microbiome data to seven factors, of which two factors showed significant differences between the three groups Supplementary Fig. S12C and S12D. Factor 1 accounted for approximately 20% of the variation and was equally contributed to by the host and microbiome; the differences between the groups were consistent those presented in Figs. 3 and 4. However, Factor 2 was exclusively contributed to by the host and revealed similarities between TT and HC.

### Cutibacterium Acnes Upregulates MYC Expression in SCC25 Oral Cancer Cells

**FIGURE 7:**
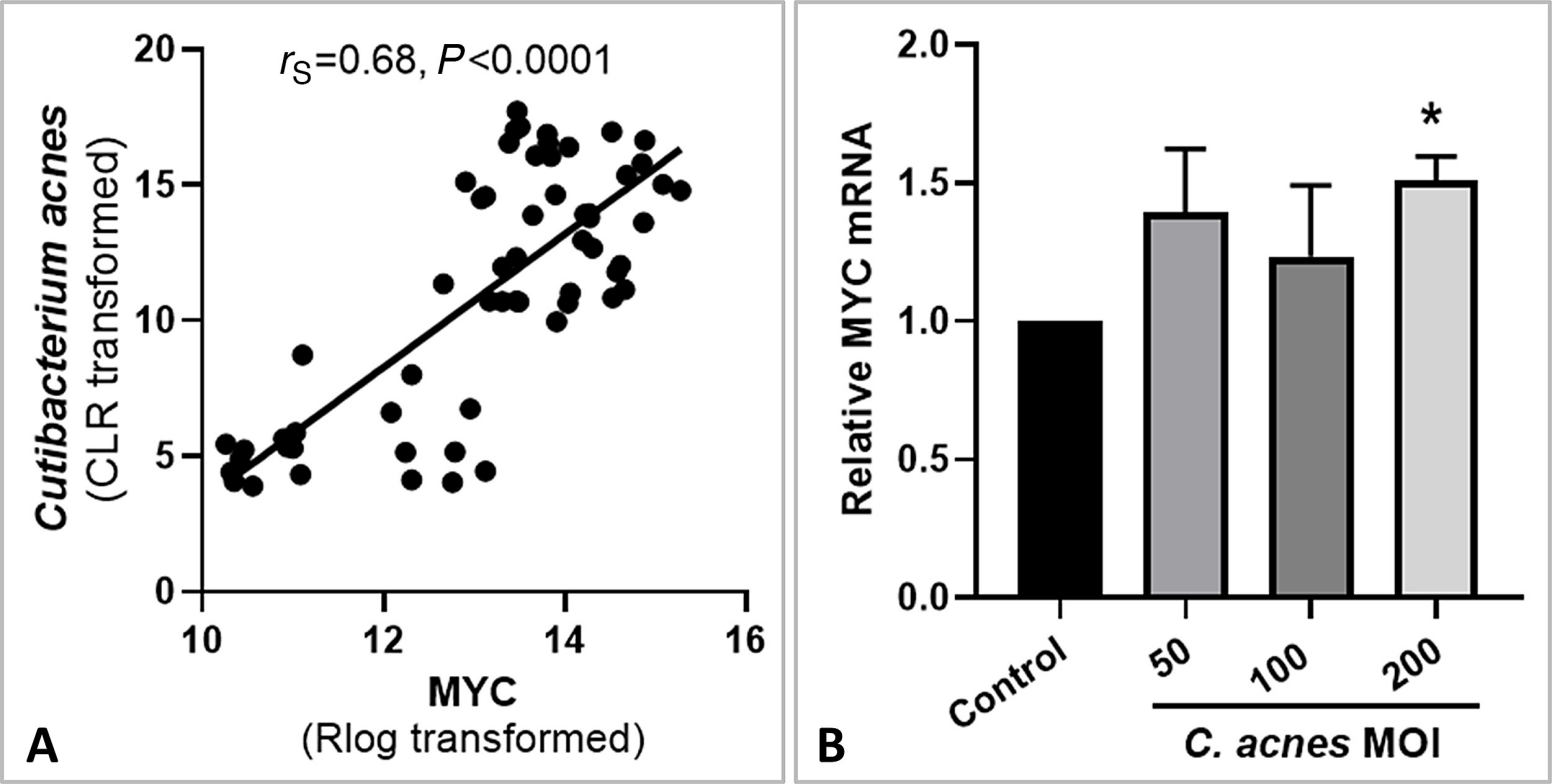
Preliminary *in vitro* experimental validation in SCC25 cells. **A,***Cutibacterium acnes* and the MYC gene were chosen for this experiment based on their strong correlation in the sequencing data. *r*_s_, Spearman correlation coefficient; CLR, centered log ratio; Rlog, regularized log transformation defined in the DESeq2 package. **B,***C. ances* NCTC 737 grown to mid-log phase was cocultured with SCC25 cells at MOI of 50, 100, and 200 for 24 hours. Levels of MYC mRNA were measured by qRT-PCR, normalized to GADPH and relative to the noninfected cells (controls). * Statistically significant, Welch-corrected *t* test.

To assess whether the observed correlations based on integrative data analysis could represent actual microbe–host cell interactions, we performed a preliminary *in vitro* validation experiment focusing on *C. acnes* and MYC gene. This pair was selected because (i) *C. acnes* was the bacteria with the highest number of significant correlations (see Fig. 6A); (ii) MYC is a key driver oncogene and was enriched as part of two of the pathways that showed association with *C. acnes*; (iii) There was a strong correlation between the two (Fig. 7A). The experiment was performed as previously described for other species (48). Briefly, *C. acnes* NCTC 737 (ATCC) grown to mid-log phase was used to infect SCC25 cells (RRID: CVCL_1682, ATCC) at multiplicity of infection (MOI) of 50, 100, or 200 for 24 hours, before the bacteria were washed and the cells used for RNA extraction. Measurement of MYC mRNA levels normalized to GADPH mRNA was performed using one-step qRT-PCR. As shown in Fig. 7B, infection with *C. acnes* resulted in upregulation of MYC expression by 1.25- to 1.5-fold, which was statistically significant at MOI of 200.

## Discussion

Using ultra-deep metatranscriptomic analysis of microdissected cancerous and adjacent normal epithelium, we identified a low abundance, yet transcriptionally active, intratumoral multi-kingdom microbiome in OSCC. LMD has been widely used in OSCC host transcriptome studies, but, to our knowledge, this is the first time it is employed to study the microbiome associated with oral cancer. For global gene expression analysis, Illumina recommends a sequencing depth of 30–60 million reads per sample. In anticipation that microbial sequences would be concealed by the highly abundant host transcripts, we performed sequencing at unprecedented 400 million paired end reads/sample (brute-force deep sequencing) which enabled us to capture the microbial transcriptome that indeed turned out to be present in very low abundance as confirmed by qPCR. Similarly, while adjacent normal tissue is an ideal control for analysis of the host transcriptome, we thought it may not be for that of the microbial metatranscriptome because the microbiome in normal tissue may be a continuum of that in the cancerous tissue. Therefore, we also included tongue scrapings from matched HC as an additional control group; a dermatologic curette was used to ensure deep epithelium sample is collected to make is as comparable as possible to the tumor samples.

Microbiome profiling identified novel findings including enrichment of *C. acnes, M. restricta, Human Herpes Virus 6B, Nupapillomavirus,* and *bacteriophages* in TT and ANT versus HC. *C. acnes* was the most transcriptionally abundant species in the TT/ANT groups (∼25 times higher than in the HC group). While *C. acnes* has not been implicated in oral cancer before, several studies have found it to be associated with prostate cancer (49–54). *C. acnes* is believed to contribute to prostate carcinogenesis through inducing chronic inflammation (55, 56), so it may play a similar role in OSCC. In this study, we found the enzyme HL to be exclusively expressed by *C. acnes* and to be significantly overexpressed in TT and ANT. HL degrades hyaluronic acid, an important component of the extracellular matrix of connective tissues. Two HLs have been characterized in *C. acnes* (57). In *Streptococcus pneumoniae*, HL is a known virulence factor involved in the spread of infection (58). Therefore, it is reasonable to hypothesize that *C. acnes* may contribute to tissue break down and thus invasion by cancer cells in OSCC via production of HLs. Further studies are required to test this hypothesis.

Other bacterial taxa less typically found in the oral cavity were associated with OSCC including known pathogens (*Moraxella catarrhalis*, and *Acinetobacter junii*) and species found typically in the skin (e.g., *Enhydrobacter Aerococcus*) or in the gut (*Cloacibacterium normanense*); while it is not clear these may play a role in OSCC, some of these species (or sister species) were found to be enriched in colorectal cancer (59). Contrary to the literature, *Fusobacterium* was not found to be associated with OSCC in our dataset, with the relative abundance being significantly higher in the tongue scrapings versus the OSCC tissues; however, *Fusobacterium nucleatum* did tend to be higher in the TT versus ANT groups (*P* = 0.1).

Viral transcripts were also enriched in the TT/ANT samples, mostly bacteriophages belonging to genus *Yuavirus* and family *Siphoviridae*. Interestingly, *Siphoviridae* have been found to be the most abundant viruses associated with colorectal cancer (60). However, their role in cancer remains not known and merits further investigation. Apart from bacteriophages, human herpesvirus 6 (HHV6) was transcriptionally more abundant, actually exclusively found, in TT and ANT, which is not entirely novel, because HHV6 has been identified in association with several types of cancer, including OSCC (61). However, unlike other herpes viruses, such as EBV and HHV8, there is no direct evidence on carcinogenicity of HHV6 (61); it is hypothesized that HHP6 may have a contributory rather than direct oncogenic role (61). Nupapilloma virus was also significantly associated with TT/ANT. This virus is represented by one species, Nupapilloma virus 1 or HPV41 (https://www.hpvcenter.se/human_reference_clones/) which has been detected in some skin carcinomas and premalignant keratosis (62), but has never been implicated in oral cancer. Finally, a small number of sequences aligned to retroviridae primarily Avian endogenous retrovirus EAV HP, which was more abundant in the TT and ANT samples. This particular species shares sequence homology with another group of viruses, Avian Leukosis Virus Subgroup J, which are known to cause diverse avian tumors (63, 64). Notably, human homolog of above virus, human endogenous retroviruses is also strongly correlated with progression of multiple tumors including squamous cell carcinoma of the head and neck (65, 66). However, possible role of avian retroviruses in human tumor samples is not known.

In a previous study using ITS sequencing, *Candida albicans* was identified as the dominant species of a dysbiotic mycobiome associated with OSCC, while *Malassezia restricta* was found to be associated with health (67). Similarly, a recent study on salivary mycobiome found a correlation between better overall survival and genus *Malassezia* abundance in patients with OSCC (68). In contrast, in this study *C. albicans* was identified in only a single sample while *Malassezia restricta* was identified frequently and was transcriptionally more abundant in the OSCC samples. One possible explanation for this apparent contradiction is that previous studies were amplicon based, that is, the species identified may have not been transcriptionally active. Indeed, in line with our findings, there is emerging evidence implicating *Malassezia* in inflammatory bowel disease as well as colorectal and pancreatic cancers (69). Consequently, the role of *Malassezia* in OSCC is worth further investigation.

To have a direct comparison between amplicon and RNA-seq–based profiles, we performed 16S RNA gene sequencing on DNA obtained from the same cells on which metatranscriptomics was carried out. While largely the same major taxa were identified by both methods, the relative abundances/rank of these taxa varied between the two methods. For *Gemella* and *Corynebacterium*, the difference was drastic. While Gemella was the most transcriptionally active genus in HC and also among the top taxa in TT/ANT, it did not feature even in top 20 most abundant genera by 16s RNA-seq. Conversely, *Corynebacterium* was among the most abundant genera in TT/ANT by 16S sequencing but found in very low abundance in the RNA-seq data. These finding demonstrate that more abundant genera may not necessarily be transcriptionally active and vice versa.

In addition to taxonomic profiling, we also obtained functional profiles in terms of enzyme classes and metabolic pathways, for which we made a few important observations. First, despite differences in taxonomic profile, the major functional features were largely similar across the three groups, which substantiates evidence for microbial functional redundancy (13). Second, the top abundant enzyme classes and pathways were related to DNA replication and transcription, response to oxidative stress and metabolism, supporting presence of a viable and transcriptionally active microbiome. Third, despite similarity in major functional groups, there were still significant differences between the TT/ANT and HC groups, including potentially relevant feature to OSCC such as HL as discussed above.

The host transcriptome in OSCC is well characterized as it has been comprehensively analyzed in several studies based on microarray and RNA-seq datasets available from The Cancer Genome Atlas (TCGA) and Gene Expression Omnibus (70, 71). A detailed comparison with results from those studies here is not feasible and largely out of the scope of the article. However, there are a few points to make. Overall, our results were consistent with the literature. For example, out of the top 25 upregulated genes and top 25 downregulated in head and neck cancer as per TCGA project [lists available from the University of Alabama at Birmingham Cancer data analysis Portal; UALCAN (72)], 49 genes were also differentially expressed in the same direction in our data. Similarly, most of the protein coding genes, lncRNAs and hub genes identified as master regulators and potential biomarkers of OSCC in recent cross-database studies (70, 73), were consistently upregulated or downregulated in our data.

A unique aspect of our study is that we also included epithelial tongue scrapings as matched controls from healthy individuals which allowed us to make three pairwise comparisons. While many of the DEGs identified in HC versus TT and ANT may not be related to the cancer process (because the samples are coming from different subjects), GSEA showed that these DEGs were enriched in several cell proliferation and cancer progression–associated pathways such as E2F targets, G_2_–M checkpoint, EMT, angiogenesis, DNA repair pathways, not only in the TT versus HC contrast but also in the ANT versus HC contrast. The latter is interesting and novel in that it indicates presence of oncogenic changes in normal adjacent epithelial tissue collected even they are not evident by histopathology evaluation, which is consistent with field cancerization (74). Understanding these potentially early oncogenic processes may have important implication for treatment of OSCC and prevention of its recurrence. Another unique aspect of our host transcriptome data is the unprecedented depth at which the samples were sequenced, which provides an opportunity for secondary analysis to identify rare transcripts and splice variant that could be playing a key role in oral carcinogenesis.

Finally, we performed integration of the microbiome and host transcriptome data to predict cancer-related host genes and/or pathways that are potentially modulated by the microbes. Given there were as many upregulated genes and microbial taxa in the HC group as there was in the TT/ANT group, one would expect, based on pure statistical associations, to see more or less equal number of gene-microbe correlations for health-associated and OSCC-associated taxa. However, we observed far more significant correlations for OSCC-associated taxa. Furthermore, only genes correlating with OSCC-associated taxa resulted in significant pathway enrichment. Together, these observations indicate the correlations identified represent potential biological interaction, not just statistical associations. Several OSCC-associated taxa, including *Cutibacterium*, Yuavirus, and Roseolovirus, showed significant correlations with more than 3,000 genes each, many of which belonged to the E2F targets, MYC targets, and G_2_–M checkpoint gene sets suggesting these taxa may contribute to carcinogenesis through interaction with proliferation pathways. Because the results based on sequencing data are highly correlative and may not necessarily reflect actual biological interactions, we performed a preliminary *in vitro* validation study in which we showed that infection by *C. acnes* upregulated expression of the oncogene MYC in SCC25 oral cancer cells, suggesting that at least some of the observed correlations are biologically valid. We are currently developing a prioritization algorithm to identify microbe-gene candidates for further validation experiments.

The study has limitations to note. First, the sample size is small, so any generalization must be done with caution. Second, given the nature of the study (i.e., analysis of sequencing data), the results are purely correlative and should be viewed only as hypothesis-generating. Third, while no RNA was detected in negative extraction control, it should have still been included in library preparation and sequencing as done with 16S analysis, to provide additional contamination control. In addition, the study would have benefited from also including a positive control (e.g., RNA/DNA extracted from a human cell line infected with a mock community). A fourth limitation is that although the controls were matched to the cases with respect to tumor site, age, sex, and ethnicity, the two groups differed in terms of lifestyle factors (namely, tobacco use and alcohol consumption) which may have confounded the results. Finally, while RNA-seq has the advantage of studying the microbial transcriptional activity, it is limited to the expressed sequences and thus does not provide information about the full composition of the microbiome in the samples.

In conclusion, to the best of our knowledge, this is the first OSCC metatranscriptomic study where the host and microbiome transcriptomes are studied simultaneously. On the host side, the study did not only confirm known oral cancer-associated genes and pathways, but also provided evidence for field cancerization by showing oncogenic changes in the adjacent normal tissue. These genes can be used as potential diagnostic markers at early stages of carcinogenesis. On the microbial side, we identified a low abundance yet unique, transcriptionally active multi-kingdom microbiome in OSCC tissues. No differences in microbiome composition between tumor/normal pairs; but marked differences compared with HC. Nevertheless, the major functional features were similar across the three groups (functional redundancy). *Cutibacterium acnes* along with its enzyme HL in addition to *Malassezia restricta*, Human Herpes Virus 6B, Nupapilloma virus, bacteriophages were key features enriched in OSCC tissues and showed potential interactions with the host transcriptome through proliferation-related pathways, which requires further validation in future mechanistic studies. Overall, this work provides novel insights into microbiome–host interaction in OSCC and opens new avenues for future microbiome research.

## Supplementary Material

Supplementary Tables 1-5 Word document with Supplementary Tables 1-5

Supplementary Figures 1-12 PDF document with Supplementary Figures 1-12

Supplementary File 1 Quantity and quality of the DNA and RNA extracts and the seqeuncing libraries.

Supplementary File 2 Detailed sequencing and mapping statistics.

Supplementary File 3 Host differenntially expressed genes

Supplementary File 4 Gene Set Enrichment Analysis results (Host pathways)

Supplementary File 5 Host differentially expressed genes and pathways by lymph node status

Supplementary File 6 Microbial relative transcriptional abundances in the samples.

Supplementary File 7 Microbial enzyme classes (EC) and metabolic pathway profiles.

Supplementary File 8 Differentially abundant microbial taxa (MaAsLin2)

Supplementary File 9 Differentially abundant microbial enzyme classes (EC) and metabolic pathways (MaAsLin2).

Supplementary File 10 Host gene-microbial genus global_correlations, rs>=0.6 and FDR=<0.05

Supplementary File 11 Host pathways (GSEA genesets) correlating with microbial genera

Supplementary File 12 Host pathways (GSEA genesets) correlating with microbial enzyme classes
