# Supplementary material for: Integrative Metatranscriptomic Analysis Reveals Disease-specific Microbiome–host Interactions in Oral Squamous Cell Carcinoma
Source: Cancer Res Commun. 2023 May 8;3(5):807–20. doi: 10.1158/2767-9764.CRC-22-0349 (PMC10166004; doi:10.1158/2767-9764.CRC-22-0349)
Supplement: Supplementary Tables 1-5 — Word document with Supplementary Tables 1-5 [file crc-22-0349-s01.docx]

**Supplementary Table 1.** Clinical characteristics of the study groups.

|  | OSCC samples  Number (%) | Healthy Control  Number (%) |
| --- | --- | --- |
| Total Sample | 20 | 20 |
| Age at the time of resection, Median (range) | 61 (44-87) | 63.5 (46-85) |
| Sex  Male  Female | 15 (75)  5 (25) | 15 (75)  5 (25) |
| Ethnicity  White  Unknown | 19 (95)  1 (5) | 19 (95)  1 (5) |
| Tumor site | Tongue | N.A (tongue scraping) |
| Lymph node metastasis  Yes  No  Unknown | 8 (40)  11 (55)  1(5) | N.A |
| Clinical Stage  Stage I  Stage II  Stage II/III  Stage III  Stage IV | 4 (20)  5 (25)  1 (5)  3 (15)  7 (35) | N.A |
| Pathological Grading  Grade I (Well differentiated)  Grade II (Moderately differentiated)  Grade III (Poorly Differentiated)  Grade IV (Undifferentiated, Anaplastic) | 3 (15)  14 (70)  1 (5)  2 (10) | N.A |
| Smoking history  Current  Previous  Never  Unknown | 7 (35)  7 (35)  4 (20)  2 (10) | None  None  20 (100)  None |
| Alcohol consumption history  Current  Previous  Never  Unknown | 11 (55)  3 (15)  4 (20)  2 (10) | None  None  None  20 (100) |

**Supplementary Table 2**: Sequences of primer sets used for microbial kingdom load determination by q-PCR.

| **Target** | **Primer name** | **Oligonucleotide sequence (5’- 3’)** | **Reference** |
| --- | --- | --- | --- |
| Bacteria 16S rRNA | 341F | CCT ACG GGA GGC AGC AG | (1) |
|  | R806 | GGA CTA CHV GGG TWT CTA AT |  |
| Archaea 16S rRNA | ARC344F | ACG GGG YGC AGC AGG CGC GA | (1) |
|  | Arch806R | GGA CTA CVS GGG TAT CTA AT |  |
| Fungal ITS | ITS1-30F | GTC CCT GCC CTT TGT ACA CA | (2) |
|  | ITS1-217R | TTT CGC TGC GTT CTT CAT CG |  |
| Human beta-actin | Actin F | CAT GTA CGT TGC TAT CCA GGC | https://pga.mgh.harvard.edu/primerbank/ |
|  | Actin R | CTC CTT AAT GTC ACG CAC GAT |  |

**Supplementary Table 3**. Validation of differentially expressed genes in RNA sequencing data using Taqman probe-based quantitative PCR assays.

| **Gene name** | **Tumor vs Normal**  **(log2 fold change)** | | **Tumor vs Healthy control**  **(log2 fold change)** | |
| --- | --- | --- | --- | --- |
|  | **q-PCR** | **RNA sequencing** | **q-PCR** | **RNA sequencing** |
| ***MMP13*** | 9.4 | 5.8 | 6.1 | 4.8 |
| ***CA9*** | 5.5 | 5.3 | 6.1 | 3.8 |
| ***ROS1*** | 3.7 | 5.3 | N.A* | 4.9 |
| ***KRT4*** | -11.0 | -6.0 | -12.7 | -5.0 |
| ***CRNN*** | -9.8 | -5.9 | -14.1 | -7.6 |
| ***SPRR3*** | -8.7 | -5.5 | -13.8 | -6.9 |

* Gene not detected in the healthy control samples by q-PCR

**Supplementary Table 4**: Primer and TaqMan probe sequences used in qPCR assay for *Cutibacterium acnes* (16S rRNA gene)

| **Primer name** | **Oligonucleotide sequence (5’- 3’)** | **Reference** |
| --- | --- | --- |
| PA-F | GCG TGA GTG ACG GTA ATG GGT A | (3) |
| PA-R | TTC CGA CGC GAT CAA CC A |  |
| PA-TaqMan probe | AGC GTT GTC CGG ATT TAT TGG GCG |  |

**Supplementary Table 5**. Grouping of top enriched viruses in the TT and ANT tissues

| Yuavirus | Bacteriophage (siphoviridae) |
| --- | --- |
| Bromovirus | Plant viruses |
| Crinivirus | Plant viruses |
| Panicovirus | Plant viruses |
| Roseolovirus | Human virus (herpesvirales) |
| Nupapillomavirus | Human virus (papillomaviridae) |
| Cucumovirus | Plant viruses |
| Potexvirus | Plant viruses |
| Sfi21dt1virus | Bacteriophage (siphoviridae) |
| Sk1virus | Bacteriophage (siphoviridae) |
| C2virus | Bacteriophage (siphoviridae) |
| P2virus | Bacteriophage (siphoviridae) |
| Alphaendornavirus | Plant/fungi host |
| Phikmvvirus | Bacteriophage (Krylovirinae) |
| Gammaretrovirus | Human virus (Retroviridae) |
| Alpharetrovirus | Human virus (Retroviridae) |

1. Takahashi S, Tomita J, Nishioka K, Hisada T, Nishijima M. Development of a prokaryotic universal primer for simultaneous analysis of Bacteria and Archaea using next-generation sequencing. PLoS One. 2014;9(8):e105592.

2. Usyk M, Zolnik CP, Patel H, Levi MH, Burk RD. Novel ITS1 Fungal Primers for Characterization of the Mycobiome. Msphere. 2017;2(6).

3. Eishi Y, Suga M, Ishige I, Kobayashi D, Yamada T, Takemura T, et al. Quantitative analysis of mycobacterial and propionibacterial DNA in lymph nodes of Japanese and European patients with sarcoidosis. J Clin Microbiol. 2002;40(1):198-204.
