# Supplementary material for: Integrative Metatranscriptomic Analysis Reveals Disease-specific Microbiome–host Interactions in Oral Squamous Cell Carcinoma
Source: Cancer Res Commun. 2023 May 8;3(5):807–20. doi: 10.1158/2767-9764.CRC-22-0349 (PMC10166004; doi:10.1158/2767-9764.CRC-22-0349)
Supplement: Supplementary Figures 1-12 — PDF document with Supplementary Figures 1-12 [file crc-22-0349-s02.pdf]

The metatranscriptomic landscape of oral squamous cell carcinoma: microbiome-host interaction

Supplementary Figures

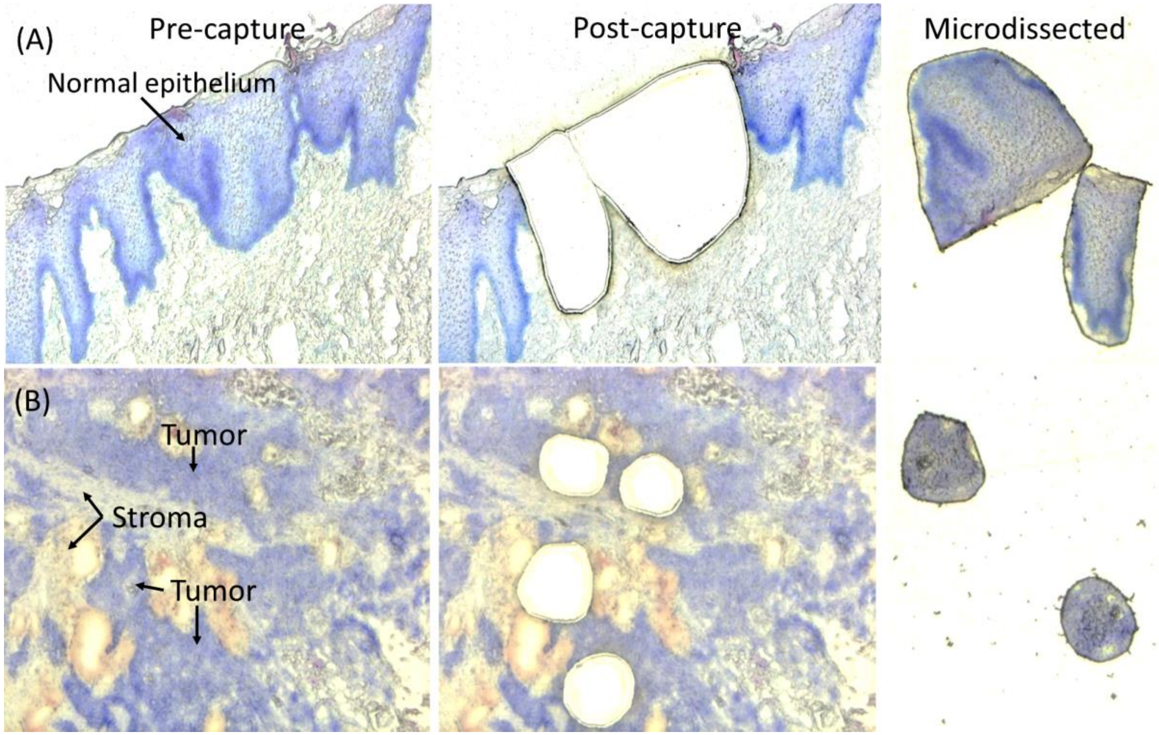

Supplementary Figure 1. Tissue microdissection. Representative images of normal (A) and cancerous (B) tissues captured with laser microdissection (LMD).

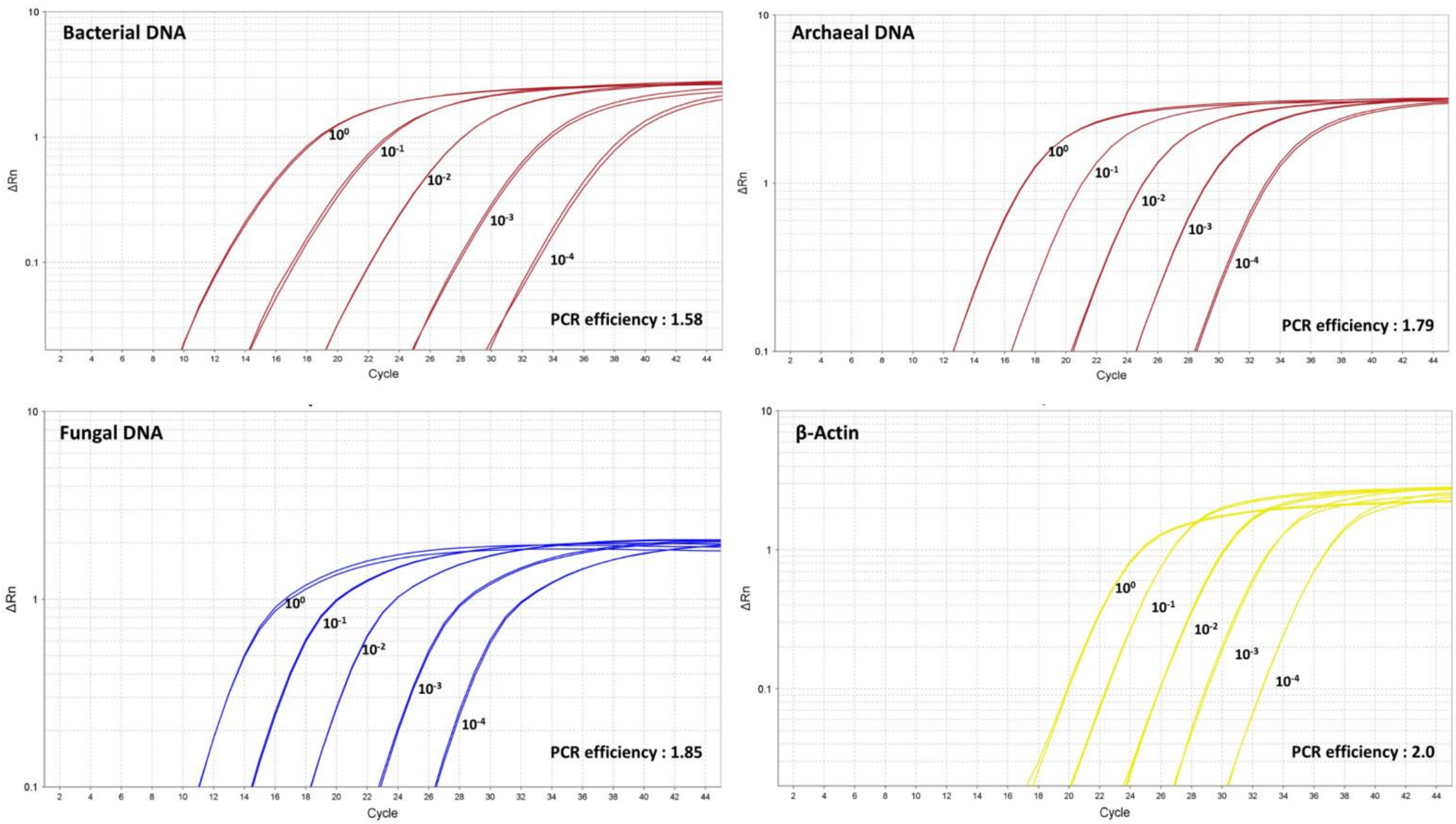

Supplementary Figure 2. qPCR standard curves. DNA prepared from *Haemophilus parainfluenzae* (NCTC 10665, Public Health England), *Methanobrevibacter oralis* (DSM 7256, DSMZ, Germany) and *Candida albicans* (CAI4 laboratory strain) for the bacterial, archaeal and fungal assays, respectively. SYBR-green chemistry was used.

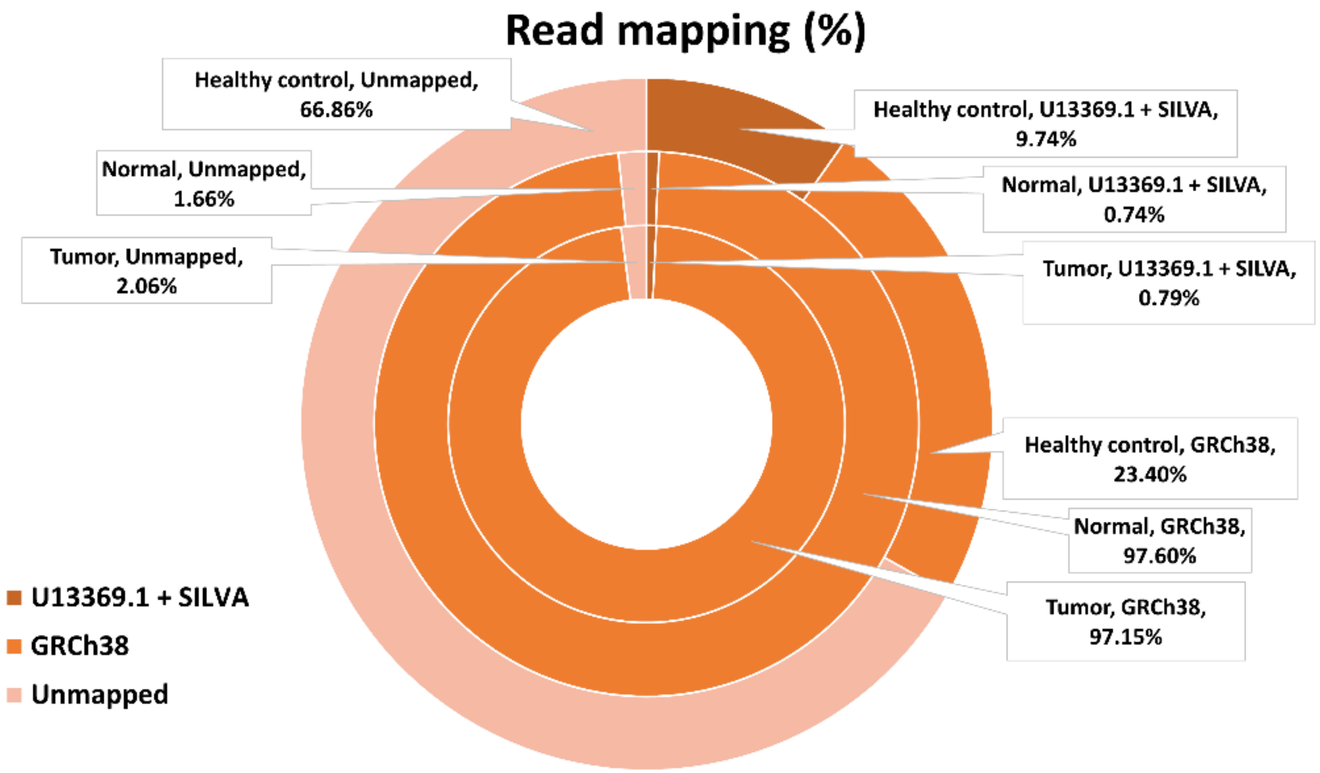

Supplementary Figure 3 . Mapping of sequencing data to the different reference databases. GRCh38, human; U13369.1, human ribosomal; SILVA, microbial ribosomal.

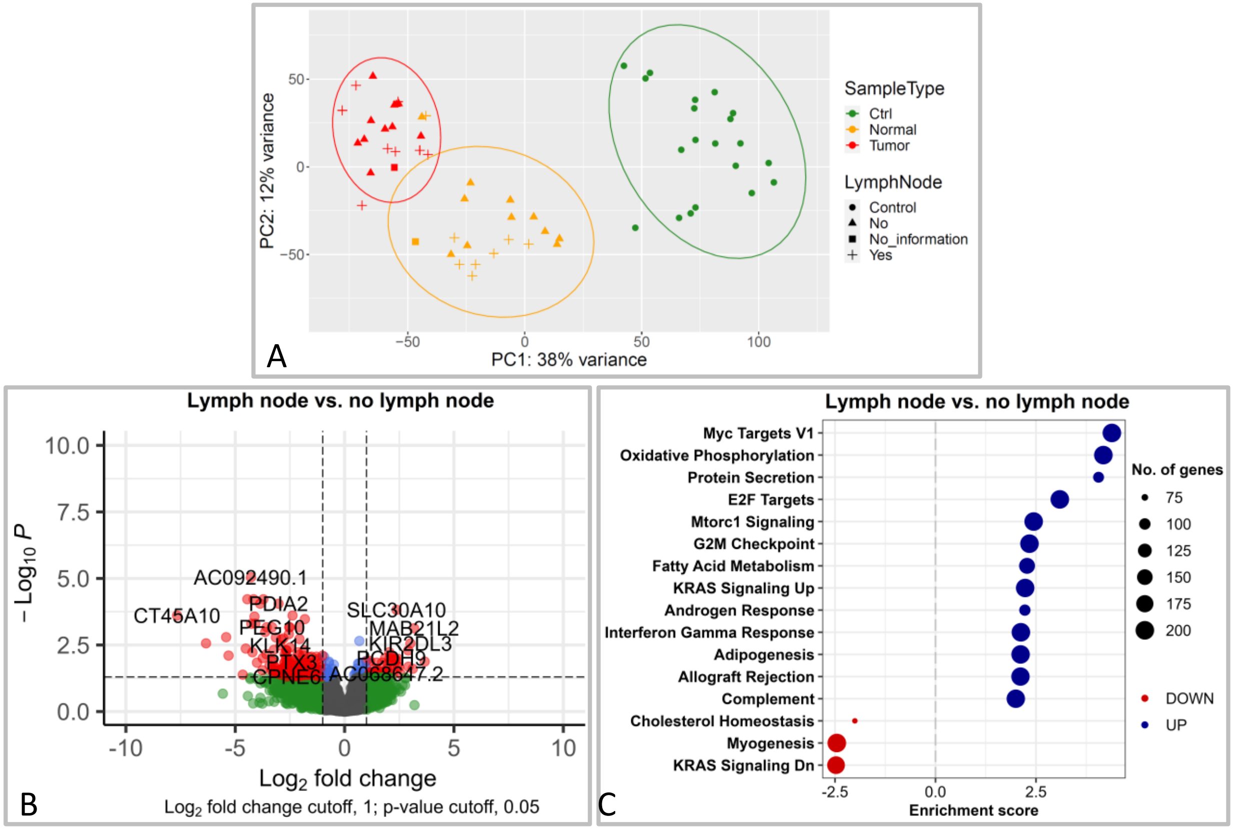

Supplementary Figure 4. Host transcriptome by lymph node involvement. (**A**) A PCA plot based on the most variable 1,500 DEGs; no clustering observed by lymph node. (**B**) A volcano plot showing DEGs in the lymph node +ve vs. lymph node –ve cases. (**C**) Upregulated and downregulated Hallmark gene sets in the lymph node +ve vs. lymph node –ve cases.

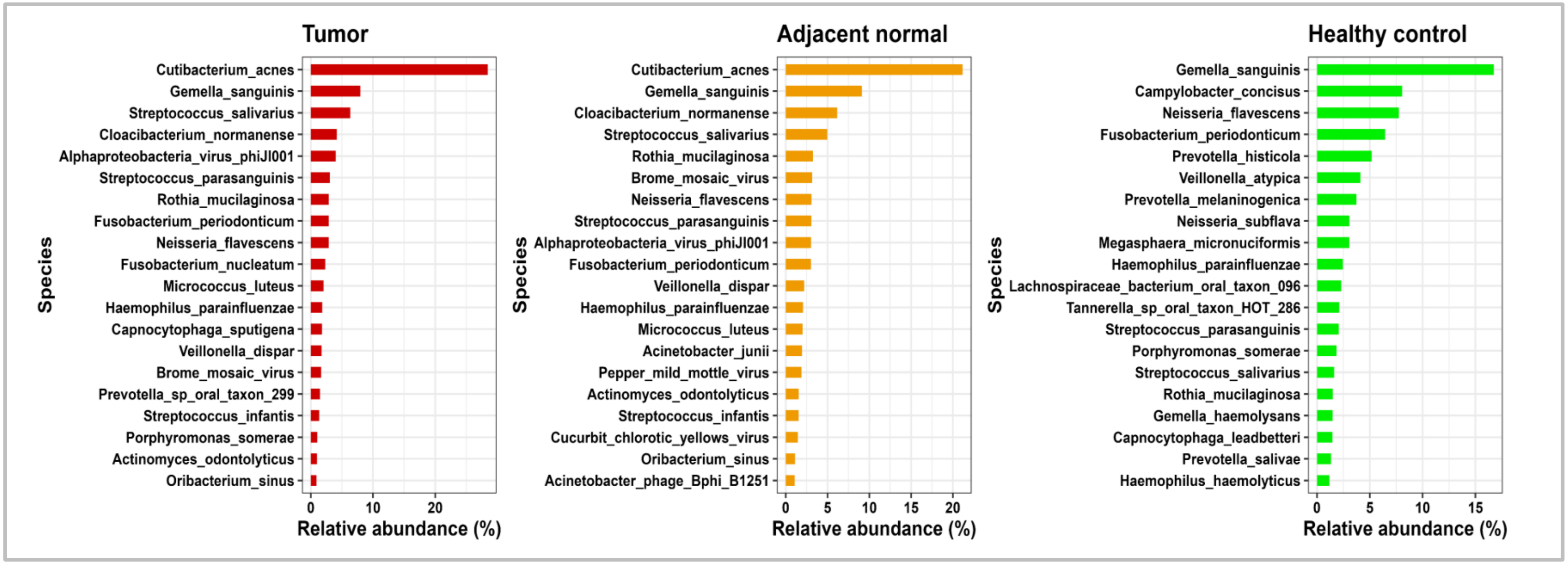

Supplementary Figure 5. Species-level transcriptional abundances. Taxonomic profiles were obtained from analysis of non-human, non-ribosomal RNA sequences with HUMAnN 3.0. The top 20 species are displayed. Compare with **Figure 4** in the main text.

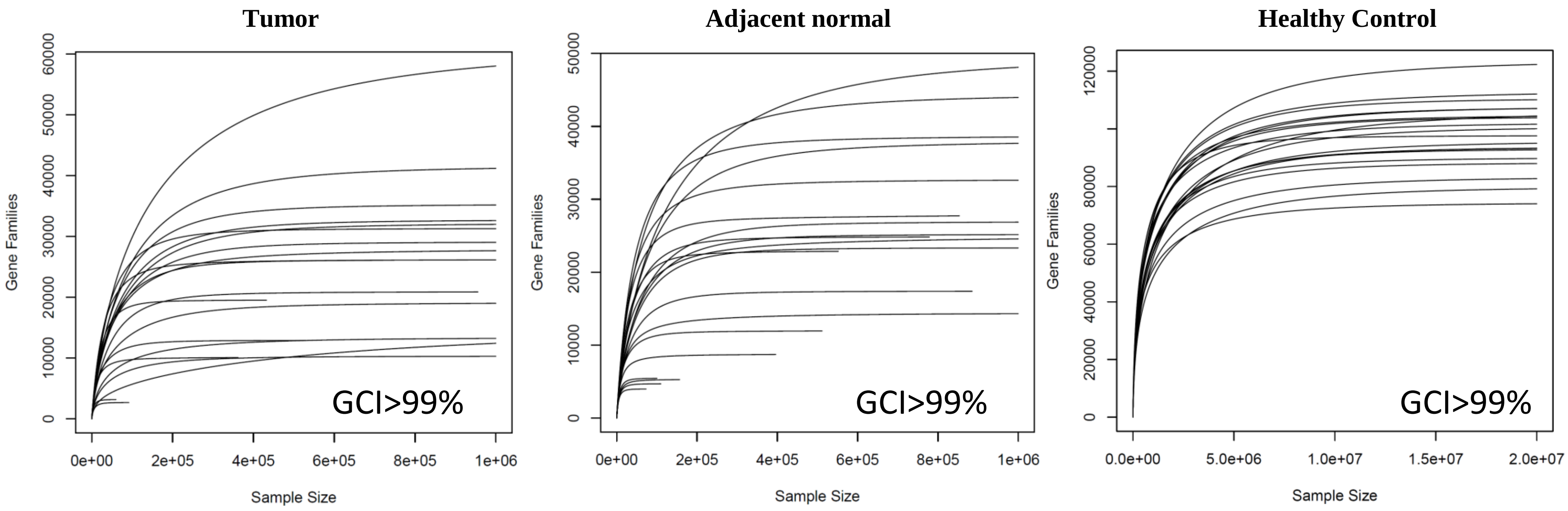

Supplementary Figure 6. Rarefaction curves. The number of microbial genes identified in each sample as a function of no. of reads successfully mapped by HUMAnN. GCI: Good's Coverage Index.

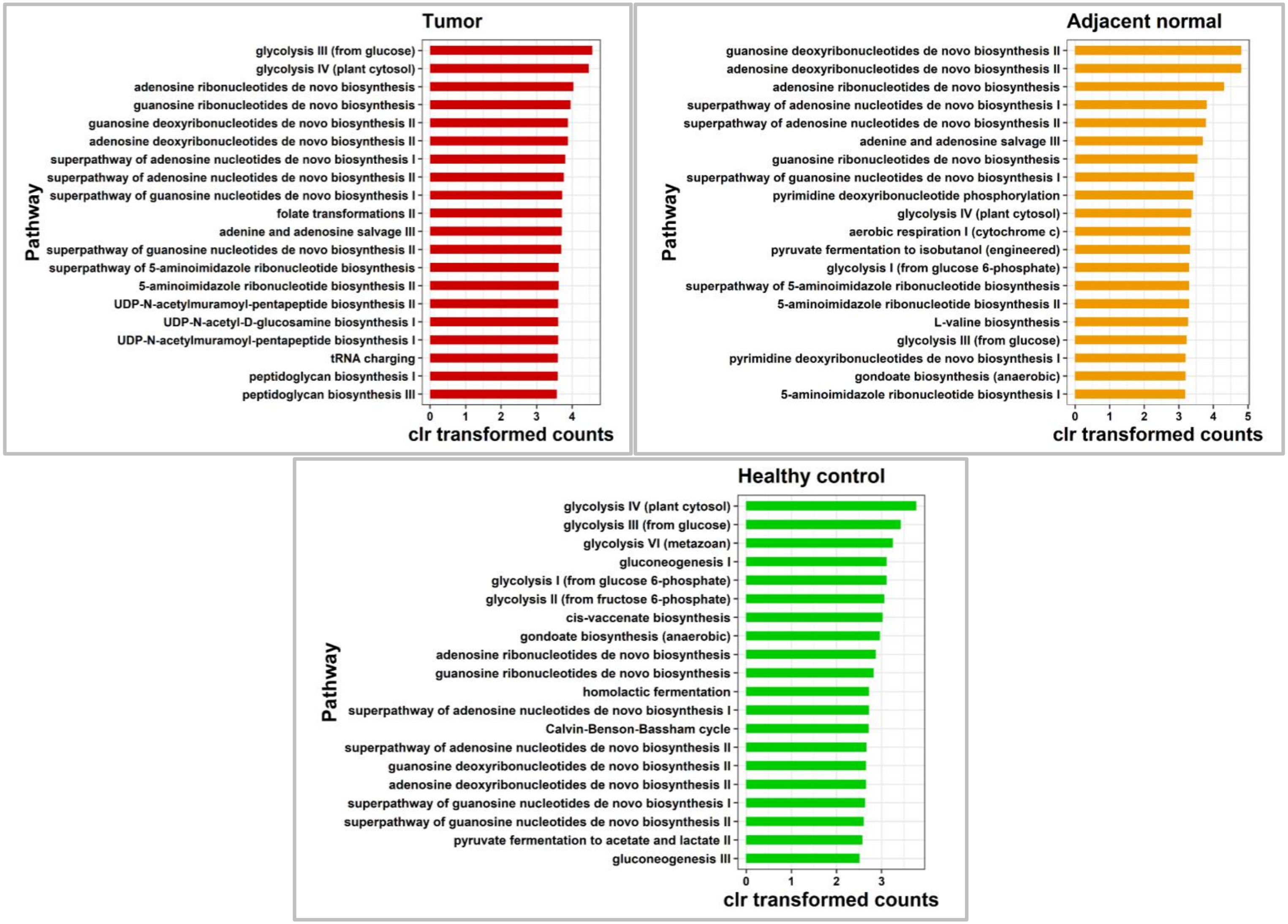

Supplementary Figure 7. Metabolic pathway-level transcriptional abundances. Functional analysis and annotation were performed with HUMAnN 3.0 using MetaCyc database . The top 20 pathways are displayed for each group.

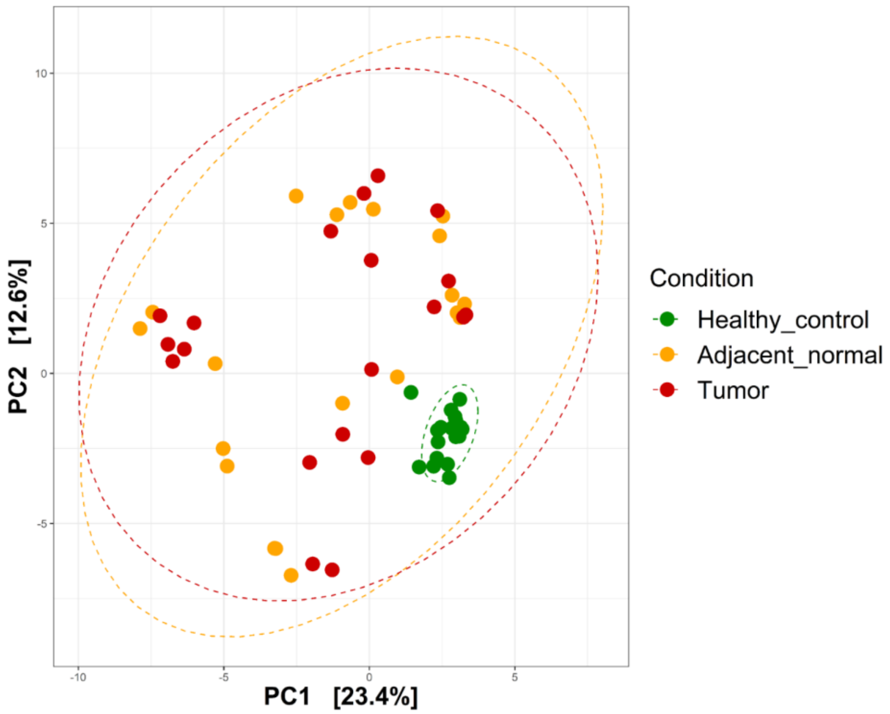

Supplementary Figure 8. A PCA plot based on CLR-transformed microbial enzyme class profiles.

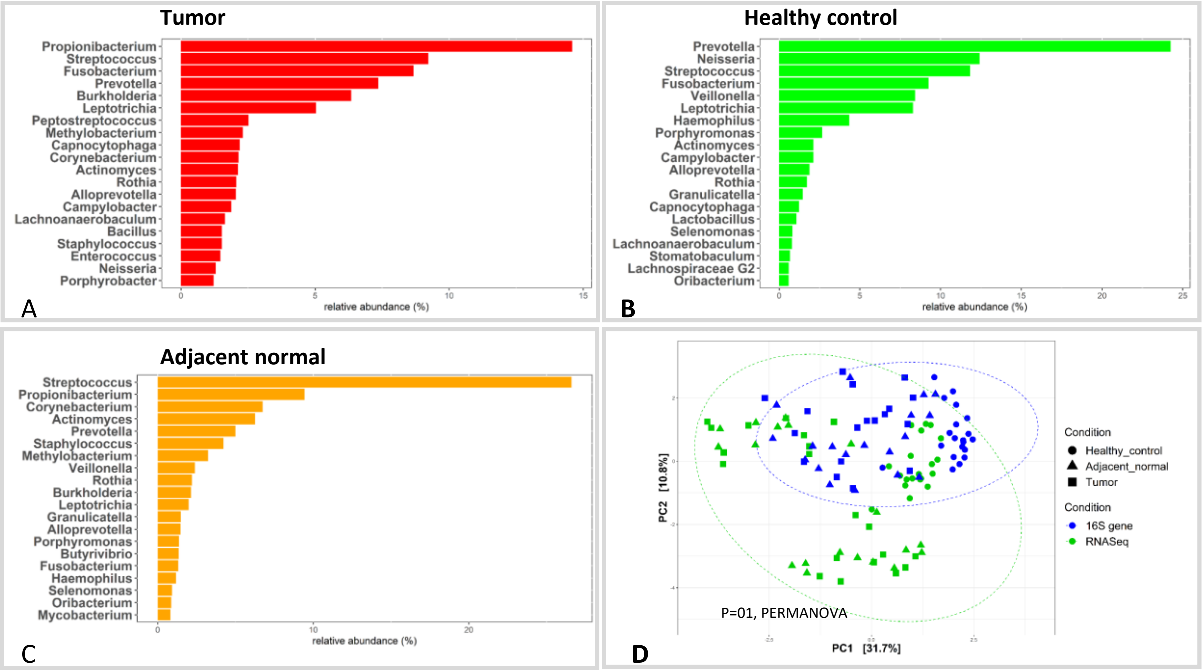

Supplementary Figure 9. **Microbial profiles by 16S sequencing**. DNA extracted from the samples was amplified for the V1-V3 region using 27FYM and 519R primers. Amplicons were sequenced on an Illumina Miseq using 2x300 paired-end chemistry. Data analysis was performed as described else where. **A-C**, the top 20 genera identified in each group. (D) A PCA plot comparing microbial profiles obtained with 16S and RNA sequencing.

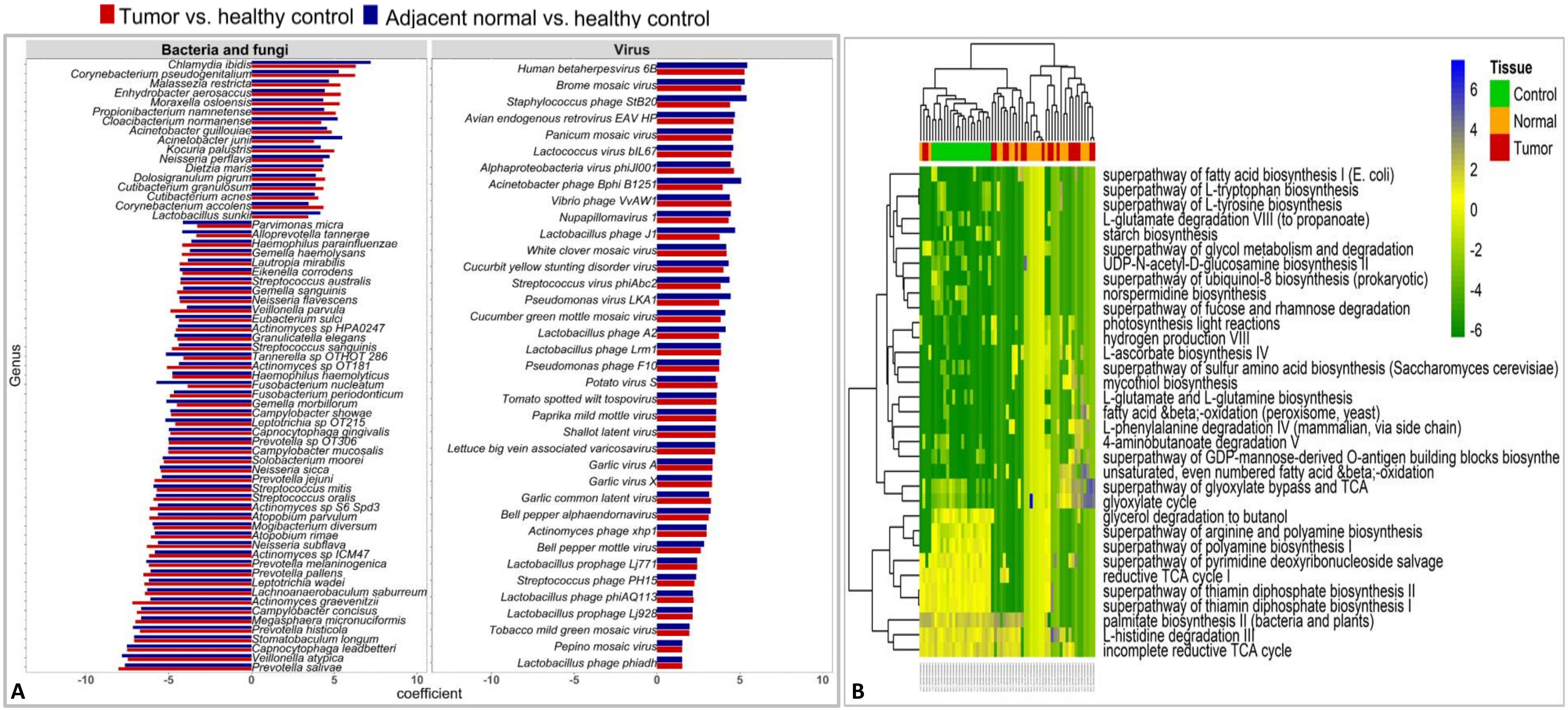

Supplementary Figure 10. Differentially abundant species and pathways. Taxonomic and functional profiles obtained with HUMAnN 3.0 were CLR transformed and differential abundance analysis was performed with MaAsLin2 setting FDR cutoff to 0.05. (**A**) Bar plots of the top differentially abundant species in the tumor vs. control and adjacent normal vs. healthy control contrasts. (**B**) A heat map showing clustering of samples based on top differentially abundant metabolic pathways.

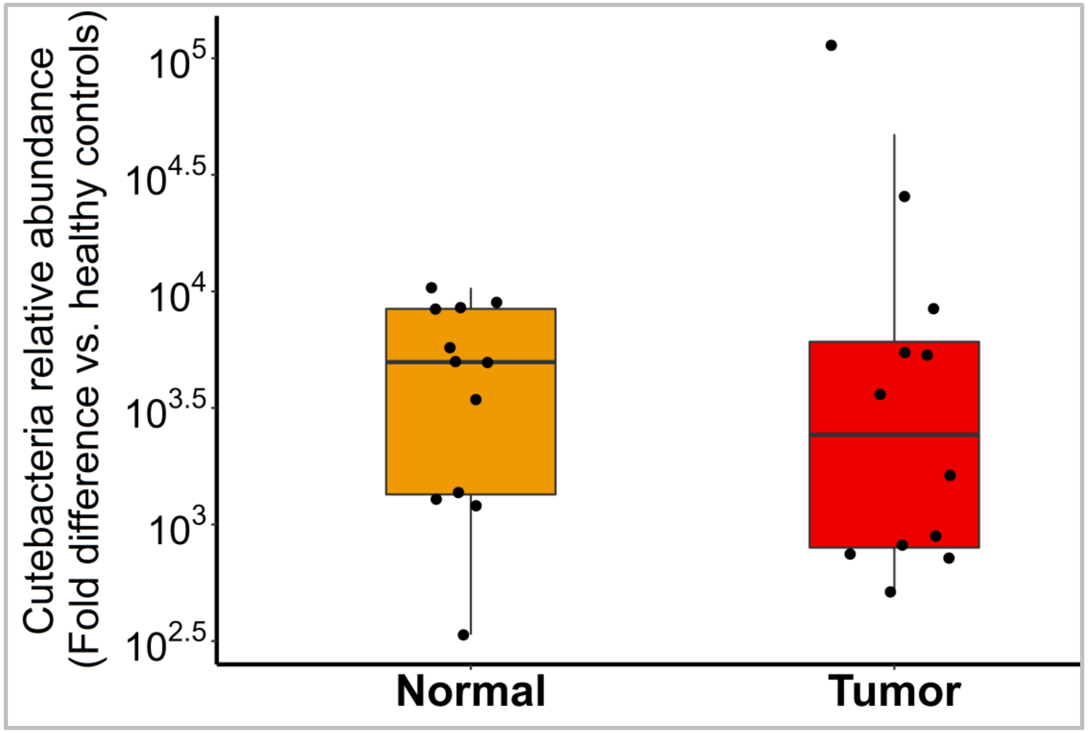

Supplementary Figure 11. Validation of differences in *Cutibacterium acnes* by qPCR. Taqman qPCR assays were performed on DNA extracted from the samples; the primer/probe sequences are listed in **Supplementary Table 3**. Abundance of *Cutibacterium acnes* was calculated relative to that of total bacteria. Data is presented in terms of fold change in relative abundance in the tumor and normal adjacent tissues compared to the healthy controls.

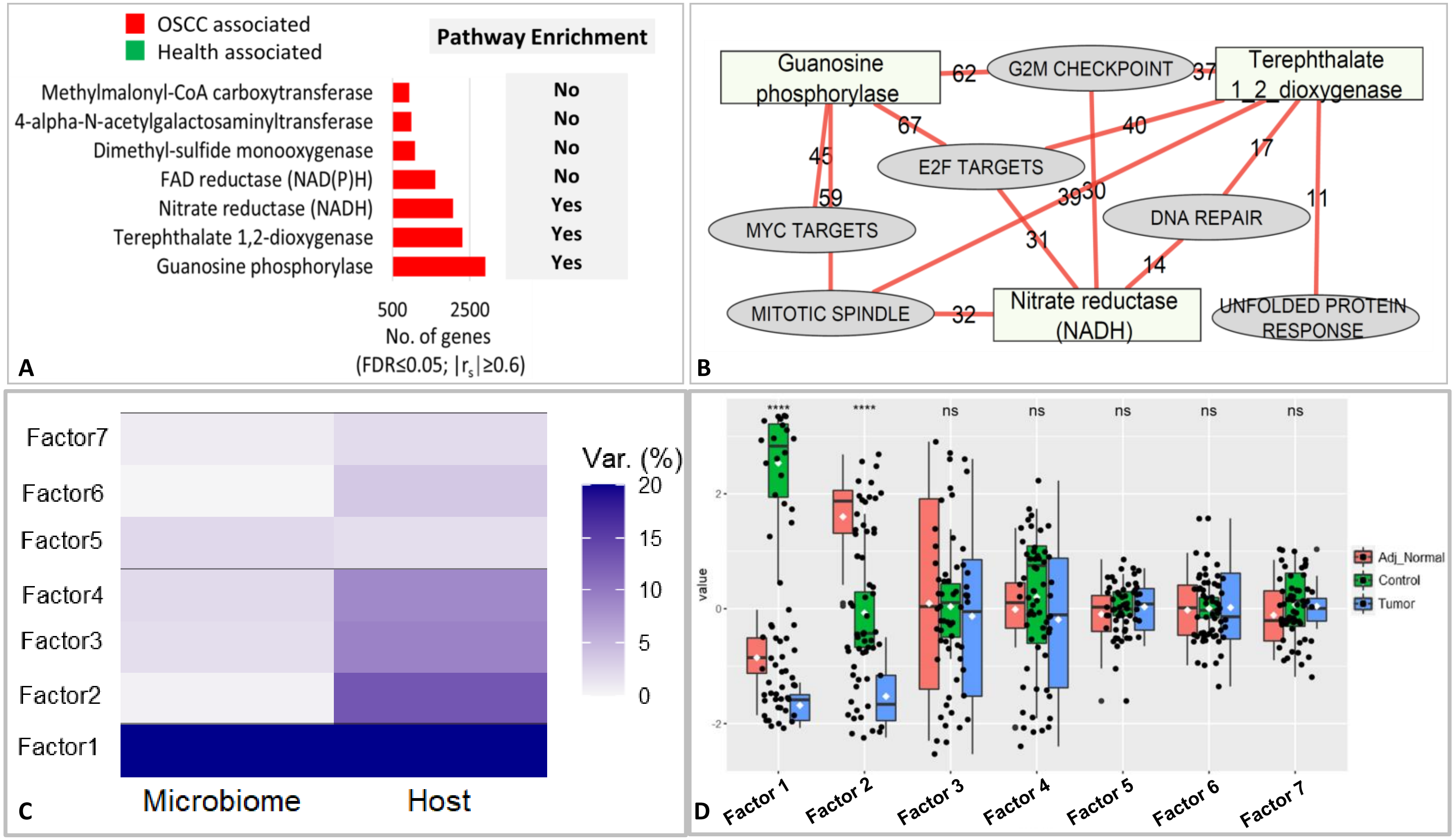

Supplementary Figure 12. Microbiome and host data integration. (A) Enzyme classes (EC) that correlated with > 500 host genes and whether GSEA turned significant results for each EC. (**B**) Interaction of selected ECs with the host pathways based on GSEA results. (**C**) Multi Omics Factor Analysis (MOFA) of microbial and host data reduced variation into 7 factors. (**D**) Factors explaining differences between the study groups.
